# Regulated cell-death programs across patient-resolved proximal-tubule states in acute kidney injury: ASPHD1-silencing increases apoptosis and lipid peroxidation

**DOI:** 10.3389/fcell.2026.1954743

**Published:** 2026-10-22

**Authors:** Zhengtong Wang, Qiaoqiao Gui, Hanjing Zhou, Xiaolei Lan, Yuan Liu, Yiyuan Zhang

**Affiliations:** 1 Department of Nephrology, Jinhua Hospital Affiliated to Zhejiang University, Jinhua, China; 2 Department of Nephrology, Rheumatology and Immunology, Chongqing University Affiliated Renji Hospital (The Fifth People’s Hospital of Chongqing), Chongqing, China; 3 Department of Emergency, The Affiliated Huai’an Hospital of Xuzhou Medical University and The Second People’s Hospital of Huai’an, Huai’an, China; 4 Department of Nephrology, The Affiliated Huai’an No. 1 People’s Hospital, Nanjing Medical University, Huai’an, China

**Keywords:** acute kidney injury, apoptosis, ASPHD1, ferroptosis, proximal tubule, regulated cell death, single-cell transcriptomics, urinary cells

## Abstract

**Background:**

Acute kidney injury (AKI) comprises heterogeneous proximal-tubule (PT) states in which regulated cell-death programs may emerge at different stages, complicating the interpretation of tissue markers and their translation to urine.

**Methods:**

Two independent human kidney transcriptomic cohorts were analyzed separately, and candidate genes were required to replicate in direction, adjusted significance, and an effect-size floor defined before candidate qualification. Candidates were localized in a patient-resolved single-nucleus kidney atlas using individual-level composition analysis and patient-state pseudobulk. Gene sets defined before correlation analysis assessed apoptosis, ferroptosis, pyroptotic inflammatory response, and programmed necrotic cell death. Urinary single-cell data examined cellular accessibility and composition, a longitudinal transplant-biopsy cohort constrained temporal interpretation, and ASPHD1 was silenced in commercial RPTEC/TERT1 cells exposed to cisplatin.

**Results:**

Nine genes met the strict cross-cohort replication criterion. ASPHD1 increased in native-kidney and transplant AKI and localized preferentially to the adaptive PT state. Apoptosis-associated transcriptional enrichment extended across adaptive and degenerative states, whereas ferroptosis-associated enrichment was most evident in degenerative PT. Candidate-to-death and death-to-immune correlations did not survive complete-family correction. ASPHD1 was detectable in urinary renal-proxy cells, but the unpaired urinary data and longitudinal transplant cohort did not establish diagnostic or recovery utility. In RPTEC/TERT1 cells, cisplatin induced ASPHD1, whereas ASPHD1-silencing reduced post-injury viability, increased annexin V-positive and cleaved-caspase-3-positive fractions, increased BAX and BBC3 while reducing BCL2, enhanced C11-BODIPY oxidation, reduced GPX4 fluorescence, and shifted SLC7A11, ACSL4, and PTGS2 in a ferroptosis-associated direction. z-VAD-FMK and ferrostatin-1 partially rescued the corresponding phenotypes.

**Conclusion:**

Human AKI PTs show state-dependent enrichment of regulated cell-death-associated transcriptional programs. ASPHD1 is an adaptive-state candidate whose silencing increases apoptotic and ferroptosis-associated vulnerability in a commercial PT injury model.

## 1 Introduction

Acute kidney injury (AKI) is a heterogeneous syndrome marked by an abrupt decline in kidney function and substantial short- and long-term morbidity (Kellum et al., 2021; Ronco et al., 2019). Tubular epithelial injury is central to many forms of AKI, yet injured proximal-tubule (PT) cells do not occupy a single molecular state: they may enter adaptive stress responses, incomplete repair programs, or degenerative states that differ in their inflammatory and remodeling context (Bonventre and Yang, 2011). The cellular origins and functional significance of bulk-tissue AKI signals, therefore, require resolution.

Regulated cell death is an organized component of kidney injury rather than an undifferentiated terminal event. Contemporary nomenclature distinguishes apoptosis, ferroptosis, necroptosis, pyroptosis, and the related modalities by their molecular execution programs (Galluzzi et al., 2018). Ferroptosis is an iron-dependent process driven by phospholipid peroxidation (Dixon et al., 2012) and can propagate synchronized tubular injury in experimental AKI (Linkermann et al., 2014), while apoptotic and necroptotic signaling can also amplify tissue damage and inflammation (Linkermann and Green, 2014). These programs may coexist, overlap transcriptionally, or arise at different stages, motivating analysis of their distribution across injured PT states.

AKI biomarker research has produced clinically informative injury and stress markers, including KIM-1/HAVCR1, TIMP2, IGFBP7, and CCL14 (Han et al., 2002; Kashani et al., 2013; Hoste et al., 2020). A transcript detected in kidney tissue, however, may have limited urinary reach because cell shedding, survival, and sample composition reshape the recovered signal. Translational qualification, therefore, requires tissue replication, cell-state localization, urinary accessibility, temporal assessment, and functional perturbation to remain distinct evidence layers.

We, therefore, combined patient-resolved, cross-compartment analysis of public human AKI datasets with a commercial human PT injury model. Candidate genes first had to change in the same direction, meet FDR <0.05, and retain a minimum absolute logFC of at least 0.30 in both the native-kidney and transplant-biopsy cohorts; they were then localized to patient-resolved PT states, related to defined death programs, placed in immune and cell–cell communication context, and examined for urinary accessibility and longitudinal behavior. This process prioritized ASPHD1 as an experimentally tractable adaptive-state candidate, after which we tested whether its silencing altered cisplatin-associated apoptosis and ferroptosis-related vulnerability in RPTEC/TERT1 cells using complementary readouts and pharmacological rescue.

## 2 Materials and methods

### 2.1 Sample information and data sources

The cohort roles, candidate rule, gene-set families, inference units, and sensitivity analyses were defined in a written analysis plan before the corresponding candidate-qualification analyses. GSE139061 contained 39 native-kidney AKI biopsies and nine nephrectomy reference samples and served as the primary tissue discovery cohort. GSE30718 contained 28 transplant biopsies with AKI and 11 pristine protocol biopsies and served as a heterogeneous tissue replication cohort (Famulski et al., 2012). GSE183277 comprised 203,702 nuclei from 10 AKI patients, 13 chronic kidney disease (CKD) patients, and 13 reference individuals and was used for individual-level cell-state localization, immune-context analysis, and CellChat analysis (Lake et al., 2023); interpretation of PT states also drew on an independent single-nuclear description of AKI states (Gerhardt et al., 2021). GSE199321 provided 40 urinary source samples from 32 patients; 29 available raw matrices were reconstructed into 27 analytic units after quality control, including 21 individual sample units from 13 patients and six pooled units, and they were used to assess urinary cellular accessibility and composition (Klocke et al., 2022). GSE126805 included 163 serial transplant biopsies from 42 recipients, of whom 37 had complete pre-implantation (PRE), post-reperfusion (POST), 3-month, and 12-month profiles, and it was used for longitudinal qualification (Cippa et al., 2018). GSE67401 contained whole-blood profiles from patients with systemic inflammatory response syndrome classified as AKI stage 0 (n = 58), stage 1 (n = 36), stage 2/3 (n = 17), or hemodialysis (n = 22), and it served as a blood-severity transfer check (Tsalik et al., 2015). GSE227970 and GSE225253 each contained three samples per condition and were retained only as commercial-cell directionality screens.

### 2.2 Bulk-tissue preprocessing and cross-cohort replication

GSE139061 expression values were mapped to approved gene symbols and retained on the processed log2 scale. GSE30718 probe-level data were processed by robust multi-array average normalization (Irizarry et al., 2003); probes were mapped through the GPL570 annotation, and the median expression of all the mapped probes was used for the gene-level matrix. A probe-level measurement audit used the same design: all probes agreed with the gene-level direction for eight of the nine strict candidates; seven of eight COL6A1 probes were positive, and one was weakly negative (logFC −0.030), while the median gene-level effect remained positive. Sample labels were checked against repository metadata, expression distributions, principal-component structure, sample–sample correlations, probe multiplicity, shared-gene coverage, mean-variance behavior, and leave-one-sample-out stability. Each cohort was analyzed separately in R 4.5.1 with data.table 1.18.2.1 and limma 3.64.3. Linear models with empirical-Bayes moderation and mean-variance trend adjustment compared AKI with nephrectomy reference tissue in GSE139061 and transplant AKI with pristine protocol biopsies in GSE30718 (Ritchie et al., 2015). Cross-cohort replication was operationally defined as concordance across these clinically heterogeneous cohorts: a gene had to be measured in both, change in the same direction, have a Benjamini–Hochberg FDR below 0.05 in each complete cohort-wide test family, and retain an absolute logFC of at least 0.30 in each cohort. The 0.30 floor was a pragmatic requirement intended to exclude statistically significant but very small cross-platform effects. Threshold sensitivity was, therefore, shown explicitly: at FDR <0.05, 13 genes passed without an effect floor, nine passed at 0.30, and four passed at 0.50. Hedges g and 95% confidence intervals summarized cross-platform effect magnitude without pooling expression values. Commercial-cell datasets and the blood-severity cohort were used for cross-compartment directionality checks.

### 2.3 Kidney single-nucleus processing and individual-level composition

The GSE183277 raw-count matrix and author-supplied metadata were aligned by barcode before analysis in R 4.5.1 with Matrix 1.7.4 and Seurat 5.4.0 (Hao et al., 2021). Library-size normalization used log [1 + counts per 10,000]. Mitochondrial and ribosomal genes were excluded from variable-feature selection; principal components were calculated with irlba 2.3.7 and two-dimensional UMAP coordinates with uwot 0.2.4 (Becht et al., 2019). UMAP was used only for visualization, and the original disease, major-class, subclass, and injury-state labels were retained; canonical markers were audited but did not replace the published annotations. Major-class proportions used all retained nuclei from the same individual as the denominator, epithelial-subtype proportions used that individual’s epithelial nuclei, and immune-subtype proportions used that individual’s immune nuclei. Total immune fraction used all nuclei from the same individual. AKI-reference abundance effects were estimated from individual-level proportions with two-sided Wilcoxon tests and 2,000 individual-level bootstrap resamples. Normalized Shannon entropy described sample mixing within subclasses.

### 2.4 Patient-level proximal-tubule pseudobulk analysis

PT nuclei were grouped from the original GSE183277 injury-state annotations as reference PT (PT-S1, PT-S2, and PT-S3), adaptive PT (aPT), or degenerative PT (dPT and dPT/DTL); these labels were inherited rather than reconstructed with a new marker classifier (Lake et al., 2023). The terms adaptive and degenerative denote cross-sectional transcriptional states. Ten AKI patients contained all three states, totaling 16,067 PT nuclei. All 30 available patient-state groups were retained; the smallest contained 66 nuclei, so no additional minimum-cell exclusion was applied. Raw counts were summed within each patient-state combination and converted to log2 counts per million plus one, generating 30 patient-state pseudobulk profiles; this patient-level aggregation preserves biological replication and avoids treating cells as independent experimental units (Squair et al., 2021; Crowell et al., 2020). Paired effects were estimated for aPT versus reference, dPT versus reference, and dPT versus aPT. Two-sided paired Wilcoxon tests were applied at the patient level and adjusted over each defined full gene family. Principal-component profiles linked the three profiles from the same patient; all-gene effect planes, candidate matrices, patient-by-gene changes, and leave-one-patient-out estimates were used to distinguish recurrent state differences from patient-specific excursions.

### 2.5 Regulated cell-death programs and candidate correlations

Four transcriptional programs were defined before candidate correlation analysis: HALLMARK_APOPTOSIS, GOBP_FERROPTOSIS, GOBP_PYROPTOTIC_INFLAMMATORY_RESPONSE, and GOBP_PROGRAMMED_NECROTIC_CELL_DEATH. Gene sets were obtained with msigdbr 25.1.1 from the Molecular Signatures Database (Liberzon et al., 2015), and pre-ranked enrichment was performed with fgsea 1.34.2 on complete gene-level statistics (Subramanian et al., 2005). Programs were labeled according to their database gene-set definitions. FDR <0.05 was categorized as supported, 0.05 to <0.10 as borderline, and ≥ 0.10 as unsupported. An orthogonal patient-level score standardized every available member gene across the 30 pseudobulk profiles and averaged those z-scores within the full gene set. For each of 12 program-by-contrast comparisons, the estimate was the mean within-patient score difference with a percentile 95% confidence interval from 5,000 patient bootstrap resamples; paired Wilcoxon P-values were adjusted across all 12 tests. Leave-one-patient-out estimates assessed influence. Exploratory candidate-to-death analysis correlated changes in ASPHD1, COL6A1, and CMTM8 with four death-program changes over three contrasts (36 Spearman tests in one correction family); death-to-immune analysis evaluated three death contrasts against six immune programs (18 tests in one family).

### 2.6 Immune-state and patient-specific communication analyses

Immune nuclei were normalized and embedded independently while retaining the published subtypes; for lineage summaries, subtypes were grouped as T/NKT, B/plasma, myeloid, dendritic, neutrophil, or mast cells. Each immune-subtype fraction used all immune nuclei from the same individual as its denominator, and patient-level abundance effects were calculated on centered log-ratio values. Six immune programs—cytotoxicity, T-cell activation/exhaustion, myeloid inflammation, phagocytic lipid metabolism, interferon response, and antigen presentation—were calculated from individual immune pseudobulk expression. AKI-reference abundance and program contrasts used individual-level Wilcoxon tests, 2,000 individual bootstrap intervals, and complete-family FDR correction. CellChat 1.6.1 was run separately for each of the 10 AKI patients with CellChatDB.human and population-size adjustment (Jin et al., 2021). Communication groups comprised reference, adaptive, and degenerative PT, T/NKT cells, B/plasma cells, myeloid/dendritic cells, fibroblasts, and endothelial cells; filterCommunication (min.cells = 20) omitted an undersized patient-specific group. A ligand–receptor pair was considered consensus-supported when inferred in at least six patients. Patient support, pathway probability intervals, source–target centrality, route structure, and leave-one-patient-out pathway ranks were retained to characterize the predicted signaling environment surrounding PT states. CellChat characterized the predicted PT-state signaling context; ASPHD1 was absent from its ligand–receptor repertoire.

### 2.7 Urinary single-cell accessibility and composition audit

Available raw matrices from GSE199321 were processed separately by library because urinary yield and RNA complexity were heterogeneous. Cells with 200–4,000 detected genes were retained; the mitochondrial-count ceiling was 20% for renal-proxy cells and 10% for non-renal proxies. Counts were converted to log [1 + counts per 10,000]. Because the available matrices did not include the authors’ cell labels, broad proxy identities were reconstructed only for composition and accessibility analyses. The renal marker set was EPCAM, CRYAB, KRT8, KRT18, KRT19, GPX3, UMOD, SLC12A1, AQP2, FXYD4, LRP2, SLC34A1, SLC4A1, and SLC26A7; the immune set was PTPRC, LST1, TYROBP, FCER1G, CD3D, CD3E, MS4A1, CD74, CSF3R, and FCGR3A; and the urogenital set was KRT13, PSCA, UPK1A, UPK2, UPK3A, KRT5, and KRT14. For each cell, a proxy score was the mean log-normalized expression of the available markers. The largest strictly positive score determined the label, with ties resolved immune over renal only when the immune score was greater than or equal to the renal score and urogenital over the other classes only when its score was greater than or equal to both; cells without a positive dominant score were classified as other/ambiguous. Repeated libraries from the same patient and sampling unit were collapsed, yielding 21 individual sample units from 13 patients plus six pools; pools were excluded from primary inference and retained only in sensitivity summaries. Candidate reach was the fraction of units with at least 1% positive cells, coverage among renal-containing units, and the renal-proxy share of positive cells. The urinary injury proxy comprised LCN2, IGFBP7, KRT8, KRT18, HAVCR1, IL32, CD40, CCL2, IFIT1, IFIT2, IFIT3, VIM, and MMP7; its cell-level value was the mean log-normalized expression of available genes, and its unit-level value was the mean among the retained renal-proxy cells. Patient-level quantities were unweighted means across that patient’s individual sample units. Across the 13 patient aggregates, the expression of ASPHD1, COL6A1, and CMTM8 was z-standardized separately. For patient i, the adaptive component was A_i = Z (ASPHD1)_i, the degenerative component was D_i = [Z (COL6A1)_i-Z (CMTM8)_i]/2, and the contrast was D_i-A_i. ASPHD1 was used because of its adaptive-PT localization; COL6A1 supplied the positive degenerative-state term, whereas CMTM8 was retained only as a non-strict contextual gene whose subtraction represented the opposite state pattern. Spearman association with the injury proxy used 5,000 patient bootstrap resamples; partial Spearman adjustment included renal-, immune-, and urogenital-proxy fractions, and unit-level, pooled-unit, and leave-one-patient-out analyses were sensitivity checks. The 13-patient analysis was exploratory, and tissue and urine samples were unpaired.

### 2.8 Longitudinal qualification and candidate selection

Within GSE126805, the complete-patient analysis included 37 recipients measured at PRE, POST, 3 months, and 12 months. Paired acute (POST-PRE) and persistent (12-month-PRE) effects were tested within patients and corrected over the defined gene family. The cohort characterized transplant ischemia-reperfusion trajectories; six-patient serial urine trajectories were descriptive because no adjudicated recovery endpoint was available. The final candidate qualification retained native-scale objectives for strict dual-tissue replication, PT-state direction consistency, urinary accessibility, and operability in a commercial PT line. Objectives were not combined into a weighted score; Pareto eligibility and objective ranks were summarized over 2,000 patient bootstrap iterations. ASPHD1 was selected for functional follow-up after the evidence-layer audit because it satisfied strict dual-tissue replication, showed positive adaptive-PT direction in all 10 informative patients, was detectable in urinary renal-proxy cells, and was amenable to intracellular perturbation in RPTEC/TERT1 cells. COL6A1 and COL7A1 remained qualified tissue candidates and led some urinary or tissue-effect objectives, but neither matched the combined adaptive-state localization and assay rationale used for the experiment. Computational summaries were generated in R 4.5.1 and Python 3.12.13 (NumPy 2.3.5, pandas 2.2.3, SciPy 1.18.0, statsmodels 0.14.6, and scikit-learn 1.9.0); NumPy and SciPy were used through their documented scientific-computing implementations (Harris et al., 2020; Virtanen et al., 2020). Figures used ggplot2 4.0.2, patchwork 1.3.2, ggrepel 0.9.6, ragg 1.5.0, Matplotlib, and Seaborn. Random seeds were 20260718 for urinary embedding and bootstrap-derived visual summaries and 20260719 for the patient-level urinary bootstrap reanalysis.

### 2.9 Cell culture

Human renal proximal tubular epithelial RPTEC/TERT1 cells (ATCC, Manassas, VA, USA; Cat. No. CRL-4031) were used in this study. RPTEC/TERT1 is an hTERT-immortalized epithelial cell line derived from the human renal proximal tubule and retains the characteristic proximal tubular epithelial properties (Wieser et al., 2008); the line has also been used for functional *in vitro* nephrotoxicity measurements (Secker et al., 2019). Cells were maintained in DMEM:F-12 Medium (ATCC; Cat. No. 30-2006) supplemented with the hTERT Immortalized RPTEC Growth Kit (ATCC; Cat. No. ACS-4007), containing the growth factors and supplements recommended for this cell line, and Geneticin™ G418 sulfate (Gibco, Thermo Fisher Scientific, Waltham, MA, USA; Cat. No. 10131035) was added at a final concentration of 0.1 mg/mL to maintain selective pressure for hTERT-mediated immortalization. Cells were cultured at 37 °C in a humidified incubator containing 5% CO_2_, and the culture medium was renewed two to three times per week. When cultures reached approximately 80%–90% confluence, cells were detached using Trypsin-EDTA for Primary Cells (ATCC; Cat. No. PCS-999-003), and enzymatic digestion was terminated with 0.1% soybean trypsin inhibitor (ATCC; Cat. No. 30-2104) before cells were collected and reseeded at an appropriate density. Cells in logarithmic growth phase were used for subsequent cisplatin injury–recovery and gene-silencing experiments. Cultures were routinely screened for *mycoplasma* contamination using the MycoAlert® PLUS *Mycoplasma* Detection Kit (Lonza, Basel, Switzerland; Cat. No. LT07-710), and only mycoplasma-negative cells were used for experiments.

### 2.10 Cell treatment, experimental grouping, and sample collection

RPTEC/TERT1 cells were seeded at an appropriate density and allowed to adhere overnight. To establish the cisplatin injury–recovery model, cells were treated with 10 μM cisplatin (MedChemExpress, Monmouth Junction, NJ, USA; Cat. No. HY-17394) or the corresponding vehicle for 24 h. At the end of the exposure period, the treatment medium was removed, cells were gently washed twice with pre-warmed phosphate-buffered saline (PBS), and fresh complete culture medium was added to initiate the recovery phase. For the injury–recovery kinetics experiments, cells were assigned to six groups: Vehicle R0, Cisplatin R0, Vehicle R24, Cisplatin R24, Vehicle R72, and Cisplatin R72. R0 samples were collected immediately after cisplatin withdrawal, whereas R24 and R72 samples were harvested after 24 and 72 h of recovery, respectively. For ASPHD1 knockdown experiments, cells were transfected with siNC, siASPHD1-1, or siASPHD1-2 approximately 24 h before cisplatin exposure and subsequently treated with vehicle or 10 μM cisplatin for 24 h. After treatment, cells were washed twice with PBS and cultured in fresh complete medium during the recovery phase. The six experimental groups were siNC + Vehicle, siASPHD1-1 + Vehicle, siASPHD1-2 + Vehicle, siNC + Cisplatin, siASPHD1-1 + Cisplatin, and siASPHD1-2 + Cisplatin. Samples were collected at the designated time-points according to the experimental endpoint. ASPHD1 knockdown efficiency, apoptosis, cell viability, and the expression of HAVCR1, COL6A1, and CMTM8 were evaluated as indicated. Three independent biological experiments were performed for each condition.

### 2.11 Quantitative real-time PCR

Total RNA was extracted from RPTEC/TERT1 cells following the indicated cisplatin treatment, recovery, or siRNA transfection using TRIzol™ Reagent (Invitrogen, Thermo Fisher Scientific, Waltham, MA, USA; Cat. No. 15596026). RNA concentration and purity were determined spectrophotometrically, and samples with an A260/A280 ratio of approximately 1.8–2.1 were used for subsequent analysis. For each sample, 1 μg of total RNA was subjected to genomic DNA removal and reverse transcription using the PrimeScript™ RT Reagent Kit with gDNA Eraser (Takara Bio, Shiga, Japan; Cat. No. RR047A). Genomic DNA was removed at 42 °C for 2 min, followed by cDNA synthesis at 37 °C for 15 min and enzyme inactivation at 85 °C for 5 s. Quantitative PCR was performed on a CFX96 Real-Time PCR Detection System (Bio-Rad Laboratories, Hercules, CA, United States) using TB Green® Premix Ex Taq™ II (Tli RNaseH Plus; Takara Bio; Cat. No. RR820A). Each 25-μL reaction contained 12.5 μL of 2× TB Green premix, 1.0 μL each of forward and reverse primers (10 μM), 2.0 μL of diluted cDNA, and 8.5 μL of nuclease-free water. Amplification was carried out at 95 °C for 30 s, followed by 40 cycles of 95 °C for 5 s and 60 °C for 30 s, and melting-curve analysis was subsequently performed to verify amplification specificity. The mRNA levels of ASPHD1, HAVCR1, COL6A1, CMTM8, BAX, BCL2, BBC3, SLC7A11, ACSL4, and PTGS2 were determined, with RPLP0 used as the endogenous reference gene. Relative transcript abundance was calculated using the 2^−ΔΔCT^ method (Livak and Schmittgen, 2001). In the cisplatin injury–recovery experiments, ASPHD1 expression was evaluated at the designated recovery time-points; for knockdown validation, ASPHD1 expression was measured following transfection with siNC, siASPHD1-1, or siASPHD1-2; and the expression of HAVCR1, COL6A1, and CMTM8 was subsequently assessed to characterize changes in tubular injury- and recovery-associated transcriptional states after ASPHD1 silencing. Three independent biological replicates were included for each experimental condition.

Primer sequences are reported 5′ to 3′, with the forward sequence followed by the reverse sequence: ASPHD1, GGA​GAG​AGA​AAG​GGG​AGA​GAA​AGG and TTC​CCT​TCC​ACA​TTC​CAC​TCT​GGG; HAVCR1, GAA​GTC​AGG​GGC​TGT​TTC​TGT​GGA and AGC​CTG​CTC​TGC​TGG​AAA​TGA​GAG; COL6A1, CGT​GGA​CCT​GTT​CTT​TGT​GCT​GGA and GTA​GTA​CCT​GTC​CCT​CAG​GTT​GTC; CMTM8, CCT​TCG​CAG​AGA​ACT​TCT​CCA​CCA and CCA​GCA​ATA​AGC​GTC​CAT​ACC​AGC; BAX, CTG​AGC​AGA​TCA​TGA​AGA​CAG​GGG and AGC​TTC​TTG​GTG​GAC​GCA​TCC​TGA; BCL2, TCG​AGC​TCT​TGA​GAT​CTC​CGG​TTG and CTT​CCC​AAT​GAA​TCA​GGA​GTC​GCG; BBC3, ATG​GAG​CCC​AAT​TAG​GTG​CCT​GCA and GGG​AGT​CCA​GTA​TGC​TAC​ATG​GTG; SLC7A11, TGG​GGT​CCT​GTC​ACT​ATT​TGG​AGC and CAC​CCA​GAC​TCG​TAC​AAA​AGC​TGG; ACSL4, TCG​CTC​TGT​CAC​ACA​CTT​CGA​CTC and TAG​GAT​TTC​CCT​GGT​CCC​AAG​GCT; PTGS2, GCA​TTC​TTT​GCC​CAG​CAC​TTC​ACG and GCT​GTC​TAG​CCA​GAG​TTT​CAC​CGT; and RPLP0, TTG​TGG​GAG​CAG​ACA​ATG​TGG​GCT and TTG​TTT​TCC​AGG​TGC​CCT​CGG​ATG.

### 2.12 Cell viability assay

Cell viability was evaluated using the Cell Counting Kit-8 (CCK-8; Dojindo Laboratories, Kumamoto, Japan; Cat. No. CK04), a water-soluble tetrazolium reduction assay used as a metabolic-activity proxy for viable-cell abundance (Stockert et al., 2018). RPTEC/TERT1 cells were seeded into 96-well plates at approximately 5 × 10^3^ cells per well in 100 μL of complete culture medium and allowed to adhere overnight. For the cisplatin dose–response experiment, cells were exposed to the indicated concentrations of cisplatin for 24 h, after which cell viability was measured to determine an appropriate concentration for establishing the injury–recovery model. For the injury–recovery assay, cells were treated with the selected concentration of cisplatin for 24 h, washed, and subsequently cultured in fresh complete medium; CCK-8 measurements were performed at the designated recovery time-points. For ASPHD1 functional analysis, cells transfected with siNC, siASPHD1-1, or siASPHD1-2 were subjected to the indicated cisplatin treatment, and cell viability was assessed at the specified time-points. At each measurement, 10 μL of CCK-8 solution was added directly to each well, followed by incubation at 37 °C for 1 h–2 h. Absorbance was measured at 450 nm using a microplate reader. Blank wells containing culture medium and CCK-8 reagent without cells were included for background correction. Relative cell viability was calculated as [(OD_sample − OD_blank)/(OD_control − OD_blank)] × 100%. Each condition was examined in triplicate wells, and three independent biological experiments were performed.

### 2.13 Immunofluorescence staining of ASPHD1

RPTEC/TERT1 cells were seeded onto sterile glass coverslips placed in culture plates and subjected to the indicated cisplatin injury–recovery treatments after reaching approximately 60%–70% confluence. At the designated time-points, cells were washed three times with phosphate-buffered saline (PBS; Gibco, Thermo Fisher Scientific, Waltham, MA, United States; Cat. No. 10010023), fixed with 4% paraformaldehyde (Sigma-Aldrich, St. Louis, MO, United States; Cat. No. P6148) for 15 min at room temperature, and permeabilized with 0.2% Triton™ X-100 (Sigma-Aldrich; Cat. No. T8787) for 10 min. After washing with PBS, non-specific binding sites were blocked with 5% normal goat serum (Gibco; Cat. No. 16210064) for 1 h at room temperature. Cells were then incubated overnight at 4 °C with a rabbit polyclonal antibody against ASPHD1 (Abcam, Cambridge, United Kingdom; Cat. No. Ab197301; 1:100). After three washes with PBS, cells were incubated with Cy3-conjugated goat anti-rabbit IgG secondary antibody (Beyotime Biotechnology, Shanghai, China; Cat. No. A0516; 1:500) for 1 h at room temperature in the dark. Nuclei were counterstained with DAPI (Beyotime; Cat. No. C1002) for 5 min, followed by three additional PBS washes. Coverslips were mounted, and fluorescence images were acquired using a confocal laser-scanning microscope under identical acquisition settings for all experimental groups. ASPHD1 fluorescence intensity was quantified from randomly selected microscopic fields using ImageJ software (National Institutes of Health, Bethesda, MD, United States) (Schne et al., 2012), with background fluorescence subtracted before analysis. Three independent biological experiments were performed for each condition.

### 2.14 siRNA transfection

Small interfering RNAs targeting human ASPHD1 (siASPHD1-1 and siASPHD1-2) and a non-targeting negative-control siRNA (siNC) were synthesized by GenePharma (Shanghai, China). RPTEC/TERT1 cells were seeded in culture plates and transfected at approximately 50%–70% confluence. siRNAs were introduced at a final concentration of 20 nM using Lipofectamine™ RNAiMAX Transfection Reagent (Invitrogen, Thermo Fisher Scientific, Waltham, MA, United States; Cat. No. 13778075). siRNA and Lipofectamine RNAiMAX were separately diluted in Opti-MEM™ I Reduced Serum Medium (Gibco, Thermo Fisher Scientific; Cat. No. 31985070), combined gently, and incubated for 10 min–15 min at room temperature. The resulting complexes were added drop-wise to the cells, which were subsequently maintained at 37 °C in a humidified atmosphere containing 5% CO_2_. Cells were harvested 48 h after transfection, and ASPHD1 mRNA expression was measured by quantitative real-time PCR to assess knockdown efficiency. Both siASPHD1-1 and siASPHD1-2 were evaluated in the initial functional experiments, and siASPHD1-2 was selected for subsequent cell-death characterization and pharmacological rescue experiments. Knockdown was verified at the mRNA level only; protein depletion was not measured.

### 2.15 Flow-cytometric analysis of apoptosis

Apoptosis was assessed using the eBioscience™ Annexin V Apoptosis Detection Kit APC (Invitrogen, Thermo Fisher Scientific, Waltham, MA, United States; Cat. No. 88-8007-72), which contains APC-conjugated annexin V, propidium iodide (PI), and annexin V binding buffer; Annexin V detects externalized phosphatidylserine, whereas PI distinguishes cells that have lost membrane integrity (Vermes et al., 1995). The 50-test kit provides annexin V-APC at 5 μL per test together with PI staining solution. Following the indicated treatments, both floating cells and adherent RPTEC/TERT1 cells were collected to minimize the loss of apoptotic cells. Cells were washed once with phosphate-buffered saline (PBS; Gibco, Thermo Fisher Scientific; Cat. No. 10010023) and subsequently with 1× annexin V binding buffer. Buffers containing EDTA or other calcium-chelating agents were avoided during staining because annexin V binding to phosphatidylserine is calcium-dependent. The cell suspension was adjusted to 1–5 × 106 cells/mL in 1× binding buffer, and 100 μL of the suspension was incubated with 5 μL annexin V-APC for 10 min–15 min at room temperature in the dark. After washing with 1× binding buffer, cells were resuspended in 200 μL of the same buffer, followed by the addition of 5 μL PI staining solution. Samples were protected from light and analyzed by flow cytometry within 4 h, in accordance with the manufacturer’s protocol. Unstained and single-stained controls were included for fluorescence compensation and gate setting, and at least 10,000 single-cell events were acquired for each sample. Flow cytometric data were analyzed using FlowJo software (BD Biosciences, Ashland, OR, united States). Annexin V-APC−/PI− cells were defined as viable cells, annexin V-APC+/PI− cells were defined as early apoptotic cells, and annexin V-APC+/PI + cells were defined as late apoptotic or secondary necrotic cells; the total apoptotic fraction was calculated as the sum of early- and late-apoptotic populations. Three independent biological replicates were performed for each experimental condition.

### 2.16 Flow-cytometric detection of cleaved caspase-3

RPTEC/TERT1 cells were transfected with siNC or siASPHD1-2 and subsequently treated with the vehicle or cisplatin at the concentration selected in the dose-optimization experiment, generating four groups: siNC + Vehicle, siASPHD1-2 + Vehicle, siNC + Cisplatin, and siASPHD1-2 + Cisplatin. After treatment, both floating and adherent cells were collected, washed twice with phosphate-buffered saline (PBS; Gibco, Thermo Fisher Scientific, Waltham, MA, United States; Cat. No. 10010023), and adjusted to approximately 1 × 106 cells per sample. Cells were fixed and permeabilized using the eBioscience Intracellular Fixation and Permeabilization Buffer Set (Invitrogen, Thermo Fisher Scientific; Cat. No. 88-8824-00), followed by incubation with PE-conjugated Cleaved Caspase-3 (Asp175) (D3E9) Rabbit Monoclonal Antibody (Cell Signaling Technology, Danvers, MA, United States; Cat. No. 12768; 1:50) for 30 min at room temperature in the dark. The cells were then washed with permeabilization buffer, resuspended in PBS, and analyzed using a flow cytometer. Cellular events were identified according to forward- and side-scatter characteristics, doublets were excluded, and PE fluorescence was recorded to determine the percentage of cleaved caspase-3-positive cells. At least 10,000 cellular events were acquired for each sample, and flow cytometric data were analyzed using FlowJo software (BD Biosciences, Ashland, OR, United States).

### 2.17 Flow-cytometric analysis of lipid peroxidation

RPTEC/TERT1 cells were transfected with siNC or siASPHD1-2 and subsequently exposed to the vehicle or 10 μM cisplatin according to the experimental design. At the indicated time-point, cells were washed with phosphate-buffered saline and incubated with 2 μM BODIPY™ 581/591 C11 (Invitrogen, Thermo Fisher Scientific, Waltham, MA, United States; Cat. No. D3861), an oxidation-sensitive lipid-peroxidation probe (Drummen et al., 2002), in serum-free medium for 30 min at 37 °C in the dark. Cells were washed, harvested, and resuspended in PBS for immediate flow-cytometric analysis. Oxidized C11-BODIPY fluorescence was detected using 488-nm excitation and the FITC channel. Cellular debris and doublets were excluded, and at least 10,000 singlet events were acquired per sample. Lipid peroxidation was quantified as the percentage of C11-BODIPY oxidation-positive cells, and data were analyzed using FlowJo software (BD Biosciences, Ashland, OR, United States). Three independent biological experiments were performed.

### 2.18 Ferrostatin-1-mediated ferroptosis rescue assays

RPTEC/TERT1 cells were transfected with siNC or siASPHD1-2 and, 24 h later, pretreated with Ferrostatin-1 (Fer-1; Selleck Chemicals, Houston, TX, United States; Cat. No. S7243; 2 μM) or an equivalent volume of DMSO for 1 h before exposure to 10 μM cisplatin (MedChemExpress, Monmouth Junction, NJ, United States; Cat. No. HY-17394). After 24 h of cisplatin treatment, cells were washed twice with phosphate-buffered saline and cultured in fresh complete medium containing the corresponding Fer-1 or vehicle during the recovery period. For cell viability analysis, cells were seeded in 96-well plates at 5 × 10^3^ cells/well, and viability was determined at R48 using Cell Counting Kit-8 (CCK-8; Dojindo Laboratories, Kumamoto, Japan; Cat. No. CK04). CCK-8 reagent (10 μL) was added to each well containing 100 μL culture medium, followed by incubation at 37 °C for 2 h, and absorbance was measured at 450 nm using a microplate reader. For lipid peroxidation analysis, parallel cultures were washed with PBS and incubated with 2 μM BODIPY™ 581/591 C11 (Invitrogen, Thermo Fisher Scientific, Waltham, MA, United States; Cat. No. D3861) in serum-free medium for 30 min at 37 °C in the dark. Cells were subsequently washed, harvested, and resuspended in PBS for immediate flow-cytometric analysis. Oxidized C11-BODIPY fluorescence was detected using 488-nm excitation and the FITC channel; cellular debris and doublets were excluded, and at least 10,000 singlet events were acquired per sample. Lipid peroxidation was quantified as the percentage of C11-BODIPY oxidation-positive cells, and flow-cytometric data were analyzed using FlowJo software (BD Biosciences, Ashland, OR, United States). Three independent biological experiments were performed for each condition.

### 2.19 Immunofluorescence staining of GPX4

RPTEC/TERT1 cells were seeded onto sterile glass coverslips and subjected to the indicated siRNA and cisplatin treatments according to the experimental design. After treatment, cells were washed three times with phosphate-buffered saline (PBS; Gibco, Thermo Fisher Scientific, Waltham, MA, United States; Cat. No. 10010023), fixed with 4% paraformaldehyde for 15 min at room temperature, permeabilized with 0.1% Triton X-100 (Sigma-Aldrich, St. Louis, MO, United States; Cat. No. T8787) for 10 min, and blocked with 5% bovine serum albumin (BSA; Sigma-Aldrich; Cat. No. A7906) for 1 h. Cells were then incubated with a mouse monoclonal anti-GPX4 antibody (Proteintech, Rosemont, IL, United States; Cat. No. 67763-1-Ig; 1:800) overnight at 4 °C, followed by incubation with an Alexa Fluor 594-conjugated goat anti-mouse IgG secondary antibody (Invitrogen, Thermo Fisher Scientific; Cat. No. A-11005; 1:500) for 1 h at room temperature in the dark. Nuclei were counterstained with DAPI (Invitrogen; Cat. No. D1306) for 5 min, and fluorescence images were acquired under identical exposure settings using a fluorescence microscope. GPX4 fluorescence intensity was quantified from randomly selected fields using ImageJ software (National Institutes of Health, Bethesda, MD, United States) (Schne et al., 2012), and the mean fluorescence intensity was used for statistical analysis.

### 2.20 z-VAD-FMK-mediated apoptosis rescue assay

RPTEC/TERT1 cells were seeded into 96-well plates at 5 × 10^3^ cells/well and transfected with siNC or siASPHD1-2. After 24 h, cells were pretreated with the pan-caspase inhibitor z-VAD-FMK (Selleck Chemicals, Houston, TX, United States; Cat. No. S7023; 20 μM) or an equivalent volume of DMSO for 1 h, followed by exposure to cisplatin (MedChemExpress; HY-17394; 10 μM) at the optimized concentration defined in the cisplatin injury model. z-VAD-FMK or vehicle was maintained during the subsequent treatment period, and cell viability was assessed at the 48-h endpoint using Cell Counting Kit-8 (CCK-8; Dojindo Molecular Technologies, Kumamoto, Japan; Cat. No. CK04). CCK-8 reagent (10 μL) was added to each well containing 100 μL culture medium, and cells were incubated at 37 °C for 2 h. Absorbance was measured at 450 nm using a microplate reader, with cell-free wells containing medium and CCK-8 reagent used for background subtraction. Each condition was examined in three independent experiments.

### 2.21 Statistical analysis

All inferential analyses used the highest available biological unit: cohort samples for bulk tissue, patients or individual samples for single-cell and pseudobulk comparisons, and independent experiments for cell assays; technical replicates were averaged and were not counted as independent observations. All tests were two-sided. Bulk differential expression used limma empirical-Bayes moderated statistics, individual-level abundance and patient-state contrasts used Wilcoxon tests as specified above, and rank associations defined in the analysis plan used Spearman coefficients. Percentile confidence intervals were obtained from 5,000 patient-level bootstrap resamples for program effects and urinary associations and from 2,000 resamples for abundance and candidate-qualification analyses. Benjamini–Hochberg correction controlled FDR within each explicitly defined test family (Glickman et al., 2014), and nominal P-values are reported only for analyses labeled exploratory. Cell-experiment analyses were performed in GraphPad Prism 10.0 (GraphPad Software, Boston, MA, United States) and are reported as the mean ± standard deviation from three independent biological experiments. Two groups were compared by unpaired Student’s t-test, single-factor experiments with more than two groups by one-way analysis of variance (ANOVA) followed by Dunnett’s test against a designated control or Tukey’s test for all pairwise comparisons, and treatment-by-time or siRNA-by-cisplatin designs by two-way ANOVA with Šidák’s multiple-comparisons test. For cell experiments, P < 0.05 was considered significant; one, two, and three asterisks denote P < 0.05, P < 0.01, and P < 0.001, respectively.

## 3 Results

### 3.1 Cohort-level quality control supports separate kidney analyses

GSE139061 comprised 39 native-kidney AKI biopsies and nine nephrectomy references, whereas GSE30718 comprised 28 transplant AKI biopsies and 11 pristine protocol biopsies (Figure 1A). Within each cohort, case and reference expression densities overlapped broadly, but the two platforms had visibly different ranges and distribution shapes (Figures 1B,C); they were, therefore, modeled separately. Principal component analysis showed phenotype-related structure together with appreciable within-group heterogeneity rather than complete case–control separation (Figures 1D,E). The strongest absolute group correlation occurred on PC3 in GSE139061 (|r| = 0.493) and PC6 in GSE30718 (|r| = 0.453), indicating that phenotype information was distributed beyond the first two components (Figure 1F). Sample-correlation matrices likewise retained cohort-specific clusters and heterogeneous within-group similarity (Figures 1G,H). The centroid-distance audit identified a more distant GSE30718 observation, with a maximum standardized distance of 6.18 versus 2.74 in GSE139061, but it did not by itself justify exclusion (Figure 1I). Probe multiplicity in GSE30718 required an explicit aggregation rule (Figure 1J): gene-level values were probe medians, and the subsequent direction audit found complete probe concordance for eight of nine strict candidates and seven of eight positive probes for COL6A1. Mapping yielded 20,139 genes in GSE139061, 22,189 in GSE30718, and 17,503 shared genes available for replication testing (Figure 1K). Cohort-specific mean-variance trends supported moderated modeling within each dataset (Figure 1L). Finally, leave-one-sample-out estimates preserved the displayed candidate directions; ASPHD1 ranged from 3.909 to 4.542 in GSE139061 and from 0.322 to 0.404 in GSE30718 (Figure 1M). The leading candidate effects were, therefore, directionally stable to the removal of individual samples.

**FIGURE 1:**
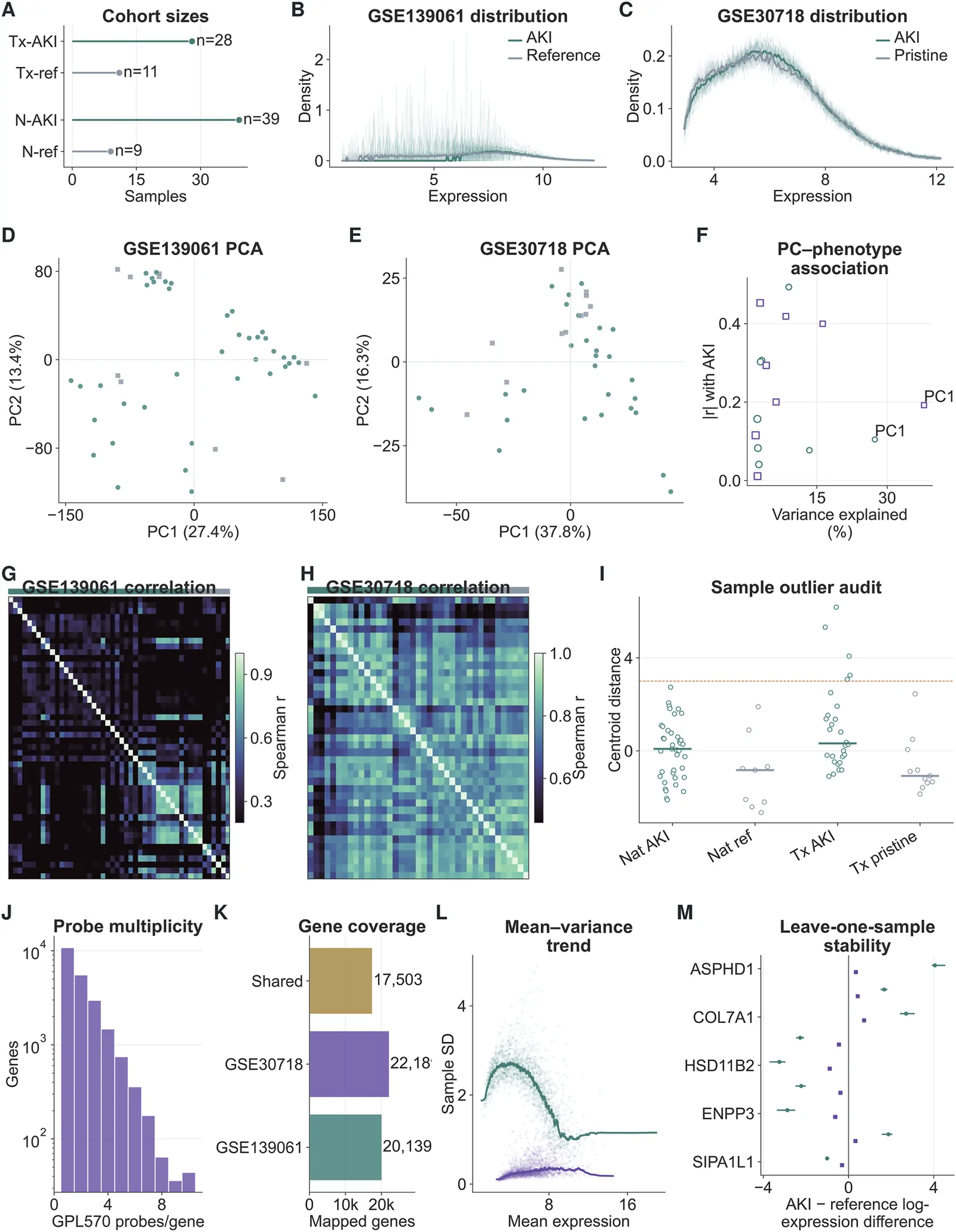
Cohort-level quality control and measurement audit for the two bulk kidney datasets. **(A)** Sample counts by cohort and group. **(B,C)** Expression distributions in GSE139061 and GSE30718. **(D,E)** Principal-component projections for each cohort. **(F)** Association between the leading principal components and disease group. **(G,H)** Sample-correlation summaries. **(I)** Distance of each sample from its group centroid for outlier assessment. **(J)** Probe multiplicity in GSE30718. **(K)** Shared and cohort-specific gene coverage. **(L)** Mean-variance relationships. **(M)** Leave-one-sample-out stability for the leading candidates. Each cohort was analyzed separately.

### 3.2 Strict cross-cohort replication identifies a nine-gene AKI tissue set

Native-kidney analysis produced a wide bidirectional AKI landscape, and ASPHD1 occupied the significantly upregulated tail (logFC 4.057, FDR 0.00569; Figures 2A,B); the leading-effect intervals illustrated the larger dynamic range of this biopsy platform (Figure 2C). Established injury markers showed mixed directions in the same tissue comparison: TIMP2 and LCN2 were positive; IGFBP7 was near zero; and VCAM1, SPP1, GDF15, and HAVCR1 were negative (Figure 2D), reflecting the specific native-kidney contrast rather than a uniform response across established markers. The transplant-biopsy cohort had smaller effect magnitudes but again contained both positive and negative signals, with increased ASPHD1 in transplant AKI (logFC 0.342, FDR 0.01776; Figures 2E–G). Across the 17,503 shared genes, effect directions were only partly concordant; the strict candidates occupied same-direction regions, whereas the background contained substantial dispersion (Figure 2H). Continuous signed-rank concordance was correspondingly weak rather than globally uniform (Figure 2I). Threshold sensitivity resolved the apparent count change: at FDR <0.05, 13 genes passed with no effect-size floor, nine remained when the minimum absolute logFC in both cohorts was at least 0.30, and four remained at 0.50 (Figure 2J). The defined strict rule, therefore, retained nine genes, while four additional genes met the two FDR cutoffs but not the 0.30 floor (Figure 2K). AGTR1, CYFIP2, and SOSTDC1 were downregulated; COL6A1, ASPHD1, HOXC8, EHBP1L1, COL7A1, and SNORA65 were upregulated. Their standardized two-cohort effects ranged from Hedges g −1.494 for AGTR1 to 1.833 for SNORA65; ASPHD1 had Hedges g 1.352 (95% CI 0.809–1.895; Figure 2L). Public commercial-cell stress datasets showed condition-dependent directions and served only as operability context (Figure 2M). Among candidates measurable in the blood dataset, none transferred to the defined severity association; ASPHD1 had rho = 0.032 and FDR = 0.900 (Figure 2N). CMTM8 was retained from this point onward only as a state-context gene, not a strict replicate, because it failed the native-kidney FDR requirement.

**FIGURE 2:**
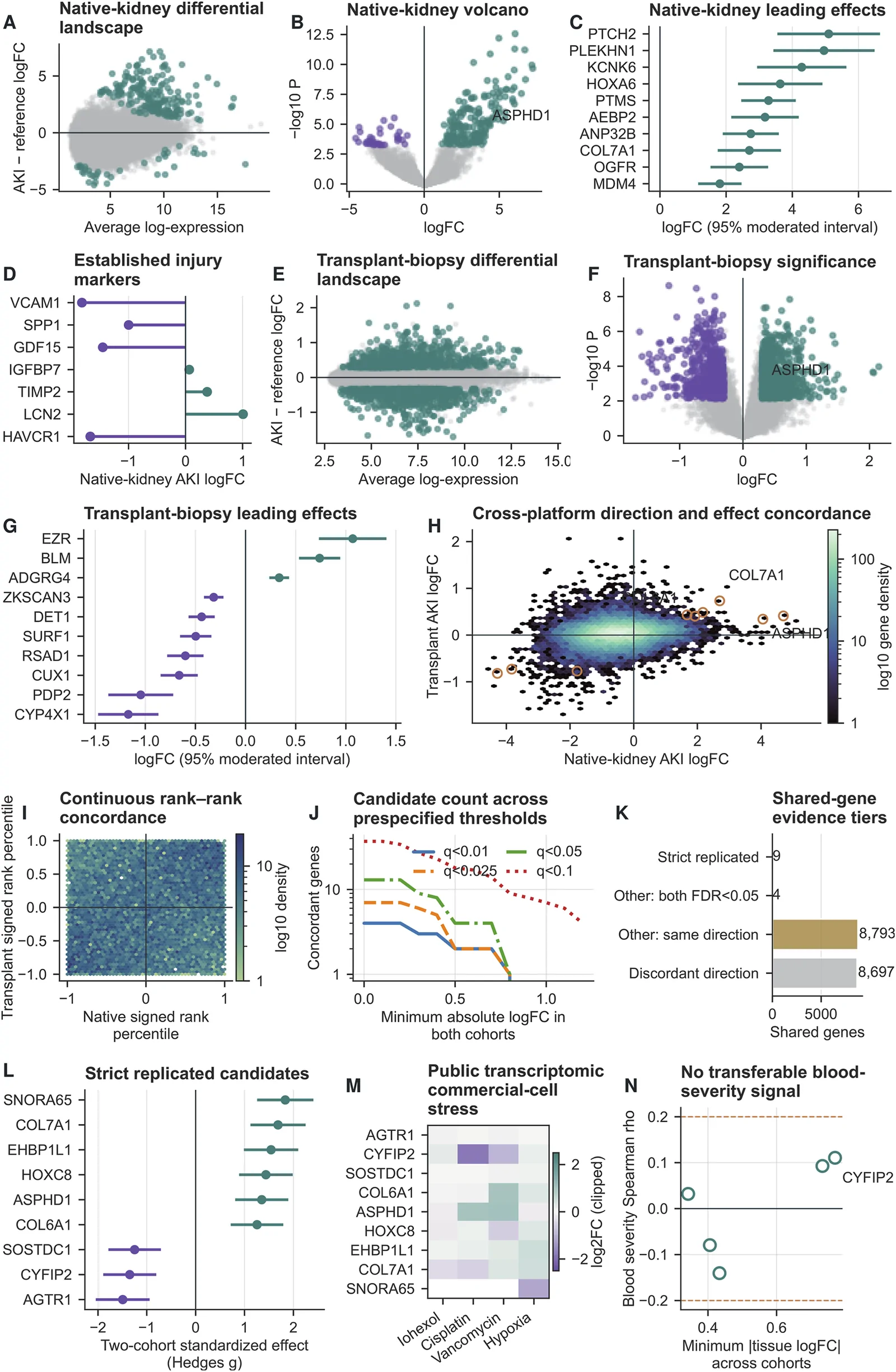
Strict cross-cohort replication identifies a nine-gene AKI tissue set. **(A–C)** GSE139061 differential-expression landscape, volcano display, and selected effects. **(D)** Standardized effects for strict candidates. **(E–G)** Corresponding GSE30718 results. **(H)** Cross-cohort effect concordance. **(I)** Continuous rank–rank concordance. **(J)** Candidate count across the defined FDR and absolute-logFC thresholds. **(K)** Shared-gene evidence tiers. **(L)** Fixed-effect standardized estimates for selected strict candidates. **(M)** Directional responses in public commercial-cell stress datasets. **(N)** Whole-blood severity-transfer check. Nine genes met the strict rule of same-direction change, FDR <0.05, and minimum absolute logFC ≥0.30 in both tissue cohorts. Public cell and blood datasets provide cross-compartment comparisons.

### 3.3 Individual-level single-nucleus analysis resolves cell sources and composition

The 203,702-nucleus atlas reproduced the published major classes and fine subclasses (Figures 3A,B). Reference, CKD, and AKI nuclei occupied overlapping regions rather than three isolated disease islands, while the retained injury-state labels showed localized enrichment within the embedding (Figures 3C,D). Sample-mixing entropy was high for several abundant subclasses, including PT (0.910) and endothelial cells (0.923), but it was lower for PapE (0.306) and ATL (0.450), indicating that some displayed populations were concentrated within fewer individual samples (Figure 3E). Individual-level heatmaps then showed substantial variation in major-class, epithelial-subtype, and immune-subtype composition within the same clinical category (Figures 3F–H). Reference-versus-AKI stromal and endothelial fractions overlapped across individuals (Figure 3I). Patient-level interval estimates included nominal changes in immune fraction (+0.026), endothelial fraction (−0.032), and principal cells (−0.066), but no tested abundance effect survived correction across the complete family (Figure 3J). Candidate-source patterns were also distinct: ASPHD1 was detectable at relatively low levels in PEC, ATL, PapE, and PT; CMTM8 was more evident in endothelial, PapE, and DCT populations; and COL6A1 was higher in IC, neural, DCT, and vascular smooth-muscle/pericyte compartments. By contrast, HAVCR1 was PT-biased, IGFBP7 was broadly expressed, LCN2 favored principal/ATL cells, and TIMP2 was prominent in podocyte, fibroblast, and endothelial compartments (Figure 3K). Canonical markers including AQP2, CALB1, COL1A1, EMCN, LRP2, MS4A1, PTPRC, SLC12A3, and UMOD showed the expected lineage-enriched patterns (Figure 3L). The marker patterns supported the retained published annotations.

**FIGURE 3:**
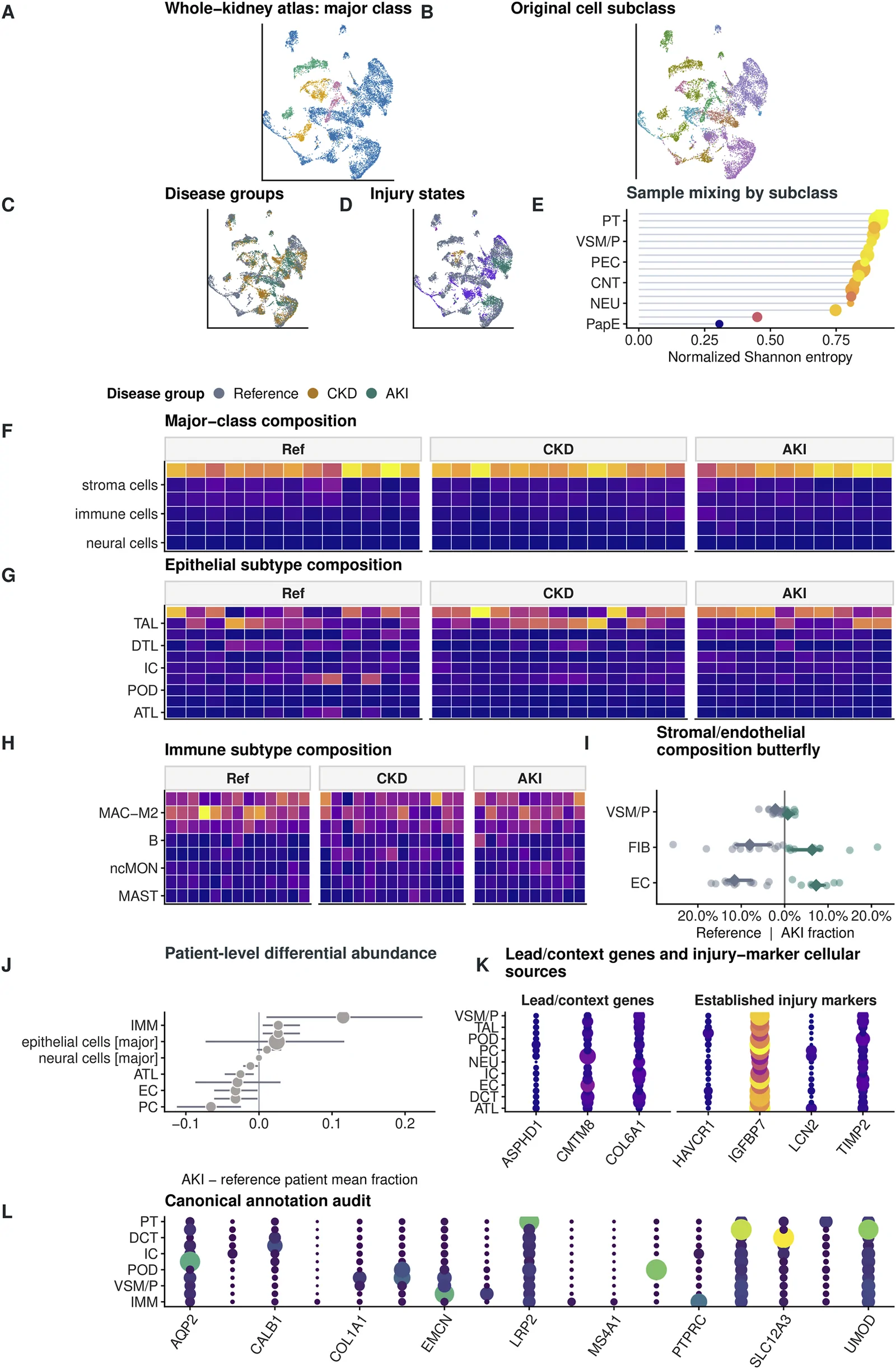
Individual-level single-nucleus kidney atlas and cellular source audit. **(A)** Whole-kidney UMAP by the major class. **(B)** Original cell subclasses. **(C)** Disease-context occupancy. **(D)** Original injury-state annotations. **(E)** Sample-mixing entropy by subclass. **(F–H)** Patient-level major-class, epithelial-subtype, and immune-subtype composition heatmaps. **(I)** Reference-versus-AKI stromal/endothelial composition butterfly. **(J)** Patient-level differential-abundance estimates. **(K)** Cellular sources of ASPHD1, COL6A1, CMTM8, and established injury markers. **(L)** Canonical marker audit of the retained annotations.

### 3.4 Patient-resolved proximal-tubule states localize candidate signals

PT-S1, PT-S2, PT-S3, aPT, dPT, and dPT/DTL occupied recognizable regions of the PT embedding and were consolidated into reference, adaptive, and degenerative states for paired analysis (Figures 4A,B). All three states contained cells from multiple patients rather than a single patient-specific island (Figure 4C). Each of the 10 AKI patients contributed one pseudobulk profile per state, although the number of contributing nuclei varied widely—from 66 to 957 for reference PT, 110 to 1,566 for aPT, and 107 to 1,157 for dPT—so pairing was performed at patient level rather than weighted by cell count (Figure 4D). Paired principal-component profiles showed variation in the direction and magnitude of state differences across patients (Figure 4E). The all-gene effect plane placed ASPHD1 in the aPT-positive region and COL6A1 toward the dPT-positive region, with established markers providing the state context (Figure 4F). In the candidate matrix, ASPHD1 was supported for aPT versus reference, COL6A1 increased in dPT versus reference, and CMTM8 and SIPA1L1 decreased in degenerative comparisons (Figure 4G). ASPHD1 generally increased from reference PT to aPT and then receded in dPT; its aPT-reference mean paired delta was 1.075 log2(CPM+1) (95% CI 0.755–1.402, FDR 0.0287), whereas dPT-reference was not supported (delta 0.326, FDR 0.381; Figure 4H). The patient-by-gene matrix retained appreciable person-to-person variation (Figure 4I). Paired estimates further localized COL6A1 to dPT (delta 0.807, 95% CI 0.368–1.317, FDR 0.0197) and CMTM8 inversely to dPT (delta −0.554, 95% CI -0.730 to −0.379, FDR 0.0197; Figure 4J). Benchmark fingerprints were not interchangeable—HAVCR1 peaked in aPT, IGFBP7 increased across both injured states, and PODXL/TIMP2 were more dPT-oriented (Figure 4K). The qualification plane, therefore, separated adaptive ASPHD1 from degenerative COL6A1 (Figure 4L).

**FIGURE 4:**
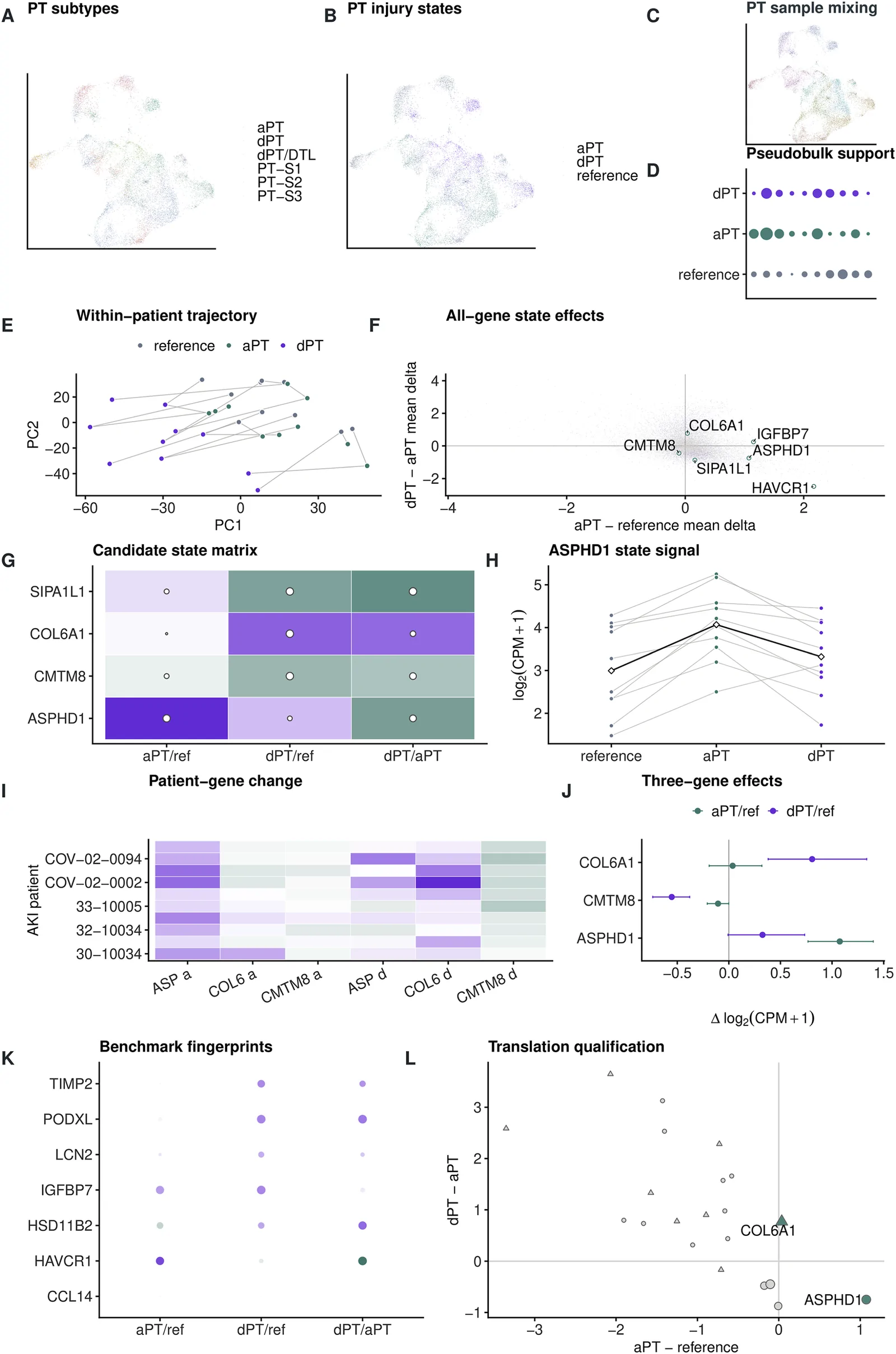
Patient-level proximal-tubule states localize the candidate signals. **(A)** PT-subtype UMAP. **(B)** Reference, adaptive, and degenerative PT states. **(C)** Patient mixing across PT states. **(D)** Patient contributions to state pseudobulk profiles. **(E)** Within-patient state profiles in principal-component space. **(F)** All-gene state-effect landscape. **(G)** Candidate state-effect matrix. **(H)** Paired ASPHD1 expression across reference, aPT, and dPT states. **(I)** Patient-by-gene change matrix. **(J)** Paired state effects for ASPHD1, COL6A1, and CMTM8. **(K)** Established-marker fingerprints. **(L)** State-localization qualification plane. Analyses use paired profiles from 10 AKI patients with all three states.

### 3.5 Regulated cell-death-associated programs differ across proximal-tubule states

The 12 defined enrichment tests separated cell-death-associated transcriptional programs across PT states (Figure 5A). The apoptosis-associated set was enriched for aPT/reference (NES 1.564, FDR 0.00214), dPT/reference (NES 2.228, FDR 2.28 x 10-11), and dPT/aPT (NES 2.086, FDR 6.84 x 10^−8^). The ferroptosis-associated set was unsupported in aPT/reference (FDR 0.667) but enriched in dPT/reference (NES 1.955, FDR 0.00259) and dPT/aPT (NES 2.161, FDR 0.000622). The pyroptotic inflammatory-response set was enriched in aPT/reference (NES 1.640, FDR 0.0250), borderline in dPT/reference (FDR 0.0567), and unsupported in dPT/aPT; the programmed-necrotic-cell-death set was enriched only for dPT/reference (NES 1.534, FDR 0.0374). The enrichment-versus-multiplicity topology, therefore, placed the strongest transcriptional evidence in degenerative-state apoptosis- and ferroptosis-associated programs rather than all modalities equally (Figure 5B). Patient-level full-gene-set scores supplied a second summary: apoptosis-associated deltas were positive for all three contrasts (0.204, 0.571, and 0.367), whereas the ferroptosis-associated aPT/reference interval crossed zero (delta 0.080, 95% CI -0.033 to 0.195), and the dPT/reference and dPT/aPT effects were larger (0.855 and 0.774; Figure 5C). FGSEA statistics and patient-score deltas gave generally concordant summaries of the same patient profiles (Figure 5D). Paired state plots showed the most consistent rise in the apoptosis-associated score across the reference–aPT–dPT ordering, a predominant ferroptosis-associated increase in dPT, and more variable pyroptotic-response paths (Figures 5E–G). Leading edges repeatedly included SQSTM1, GPX4, NINJ1, CASP3, CASP1, BAX, CASP7, CASP4, TNF, and PYCARD, with several genes shared across programs rather than unique to one modality (Figure 5H). Leave-one-patient-out estimates retained the principal positive apoptosis- and ferroptosis-associated directions (Figure 5I). None of the 36 candidate-to-death correlations passed complete-family correction (Figure 5J), and none of the 18 death-to-immune correlations did so either (Figure 5K).

**FIGURE 5:**
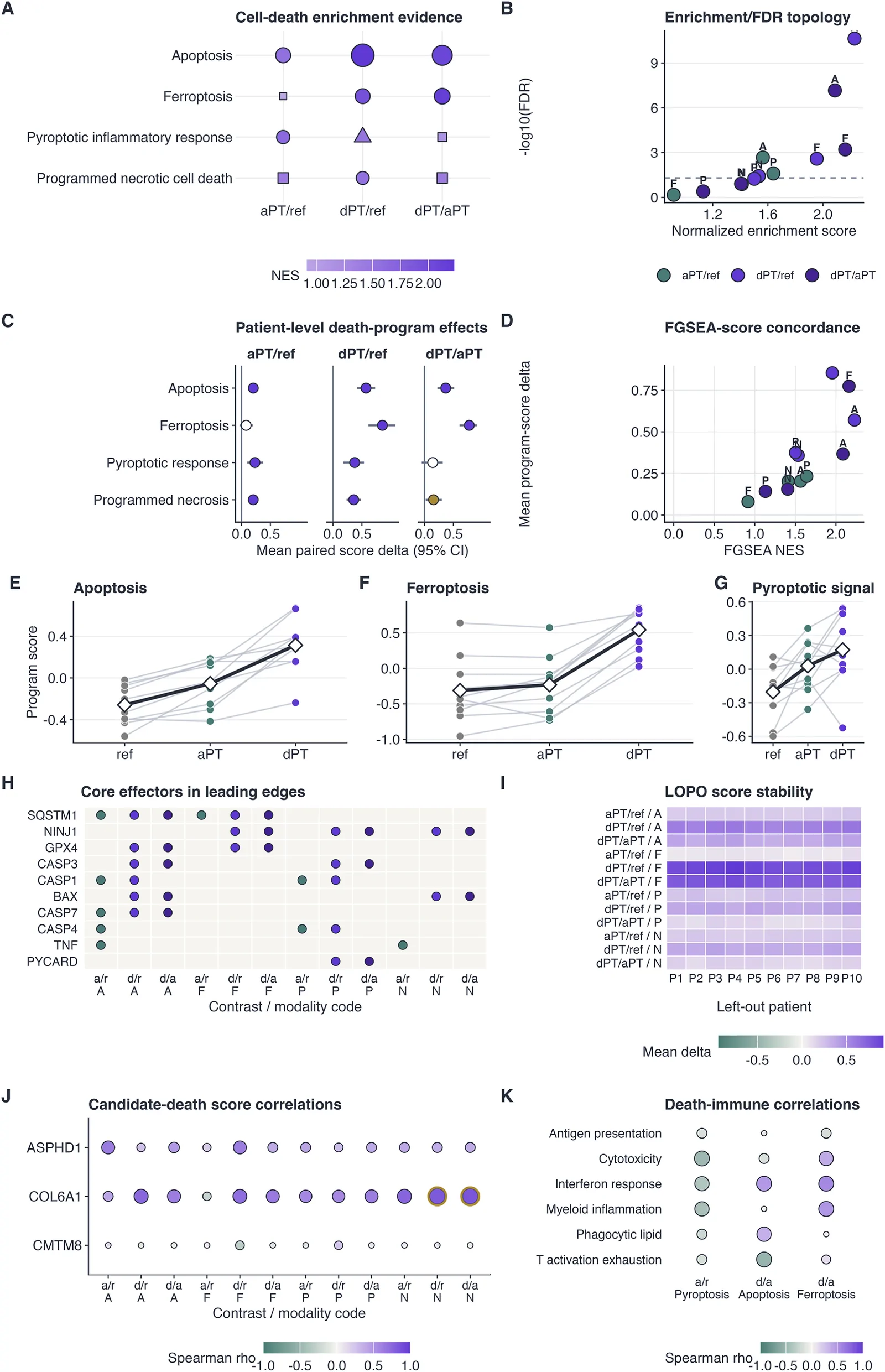
Regulated cell-death-associated programs differ between adaptive and degenerative proximal-tubule states. **(A)** Complete FGSEA evidence for the four gene sets defined before candidate-correlation analysis across aPT/reference, dPT/reference, and dPT/aPT contrasts. Color indicates normalized enrichment score, symbol size indicates enrichment magnitude, and symbol shape indicates the FDR evidence class. **(B)** Enrichment-versus-FDR topology. **(C)** Mean within-patient full-gene-set score differences with 95% confidence intervals from 5,000 patient bootstrap resamples. **(D)** Concordance between FGSEA NES and patient-level score effects. **(E–G)** Patient-resolved apoptosis-, ferroptosis-, and pyroptotic-response scores. **(H)** Core death and inflammatory effectors among leading edges. **(I)** Leave-one-patient-out stability. **(J)** Candidate-to-death-score correlations. **(K)** Death-to-immune-program correlations. Panels C and E–I use the 10 AKI patients with all three PT states; patient is the biological unit. Paired Wilcoxon tests were adjusted over the 12 program-by-contrast tests. Panels J and K are exploratory, and none of their correlations passed complete-family Benjamini–Hochberg correction.

### 3.6 Immune heterogeneity limits average state effects

The immune embedding retained 12 published subtypes, and the reference, CKD, and AKI cells occupied several shared subtype regions rather than an AKI-exclusive immune cluster (Figures 6A,B). T cells accounted for the largest fraction overall; AKI patients showed lower displayed MAC-M2 and higher ncMON/PL fractions than reference individuals, but these were descriptive patient-level shifts (Figure 6C). Total immune fraction was higher in AKI by a median 0.023, with an individual-level bootstrap interval of 0.004–0.040 (Figure 6D), while the subtype heatmap showed pronounced within-condition heterogeneity, particularly for MAC-M2 (Figure 6E). Across the complete subtype family, no abundance contrast survived FDR correction; the largest nominal pattern was lower MAC-M2 abundance (effect −0.673, P = 0.020, and FDR = 0.240; Figure 6F). Immune-program heatmaps, likewise, showed patient-specific combinations rather than one uniform AKI profile (Figure 6G), and none of the six program contrasts passed correction; interferon response had an effect of 0.447 with nominal P = 0.0438 and FDR = 0.263 (Figure 6H). Candidate and benchmark transcripts arose from different immune lineages (Figure 6I). These results show inter-individual immune heterogeneity without an adjusted group-level subtype or program effect.

**FIGURE 6:**
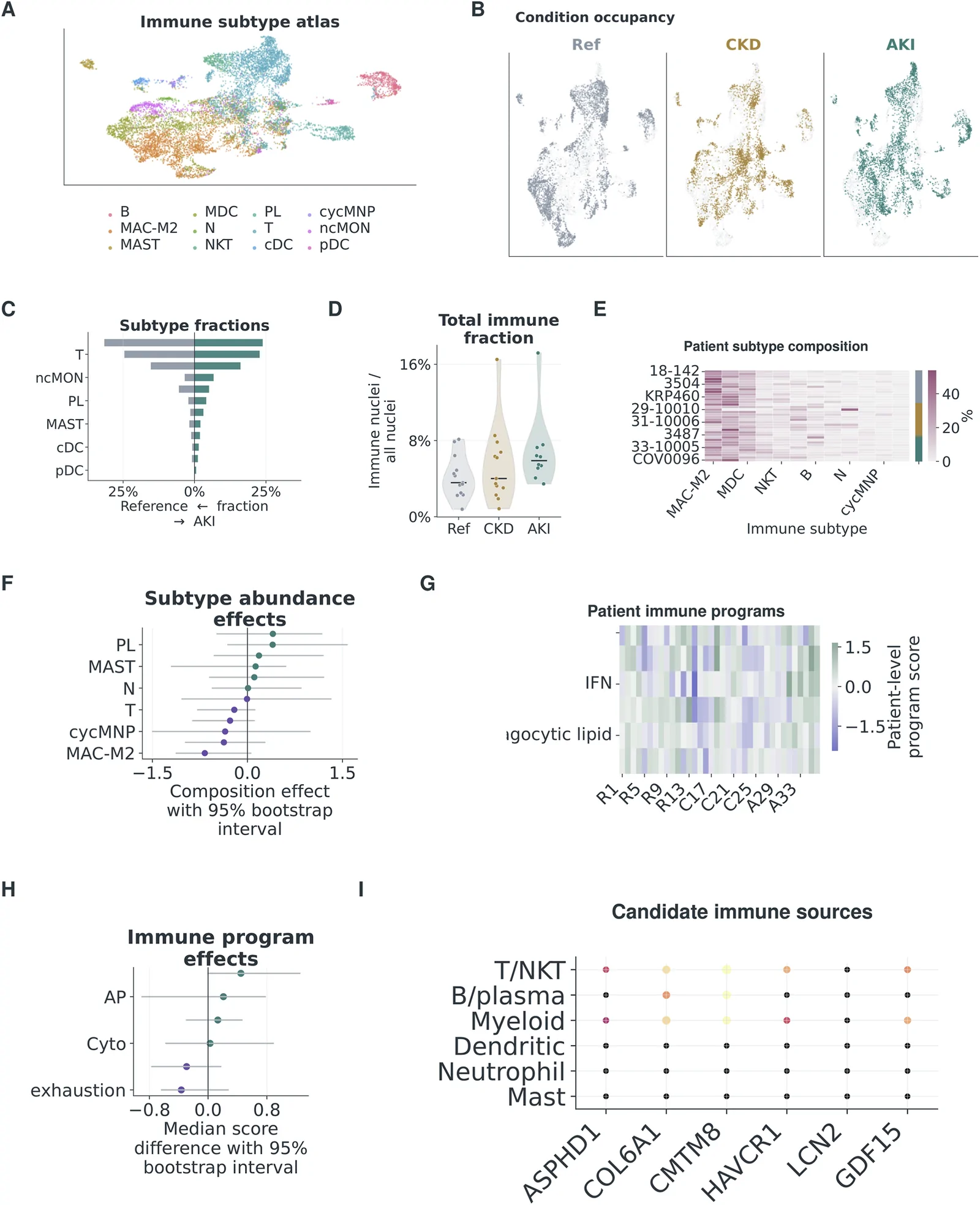
Immune-state variation defines the inflammatory context of AKI. **(A)** Immune-subtype UMAP. **(B)** Reference, CKD, and AKI occupancy. **(C)** Reference-versus-AKI subtype fractions. **(D)** Total immune fraction by condition. **(E)** Patient-level immune-subtype composition. **(F)** AKI-reference subtype-abundance effects with 95% individual-bootstrap confidence intervals. **(G)** Patient-level immune-program scores. **(H)** AKI-reference immune-program effects with 95% bootstrap confidence intervals. **(I)** Immune-lineage sources of selected candidate and benchmark genes. GSE183277 included 10 AKI patients, 13 CKD patients, and 13 reference individuals; individuals, not nuclei, are the biological units. Abundance and program contrasts used two-sided Wilcoxon tests and complete-family Benjamini–Hochberg correction. No immune subtype or program passed correction.

### 3.7 Patient-specific communication defines recurrent tissue context

Communication groups were not equally available in all 10 AKI patients, and groups with fewer than 20 cells were omitted from the corresponding patient model (Figure 7A). The source–target matrix placed aPT among the highest CellChat-estimated communication-strength groups (Figure 7B), but support across patients was uneven for many group pairs (Figure 7C). Pathway intervals highlighted COLLAGEN, PTPRM, LAMININ, SPP1, NRG, and APP contexts, with median modeled weights of approximately 0.119 for COLLAGEN, 0.093 for PTPRM, 0.041 for LAMININ, and 0.038 for SPP1 (Figure 7D). aPT had the highest displayed median outgoing and incoming centrality (0.143 and 0.157), while fibroblast and endothelial groups also occupied visible sender or receiver positions (Figure 7E). Consensus routes inferred in at least six patients were concentrated around aPT, including aPT-to-aPT and fibroblast-to-aPT collagen, aPT/dPT-to-aPT SPP1, and aPT/fibroblast-to-aPT laminin predictions (Figure 7F). APP, LAMININ, PARs, and VISFATIN pathway ranks were relatively insensitive to the removal of one patient (Figure 7G). Recurrent predicted SPP1 edges spanned several senders and receivers (Figure 7H), and leading extracellular-matrix hypotheses included COL4A1-(ITGAV + ITGB8) from aPT to aPT and SPP1-integrin combinations directed toward aPT (Figure 7I).

**FIGURE 7:**
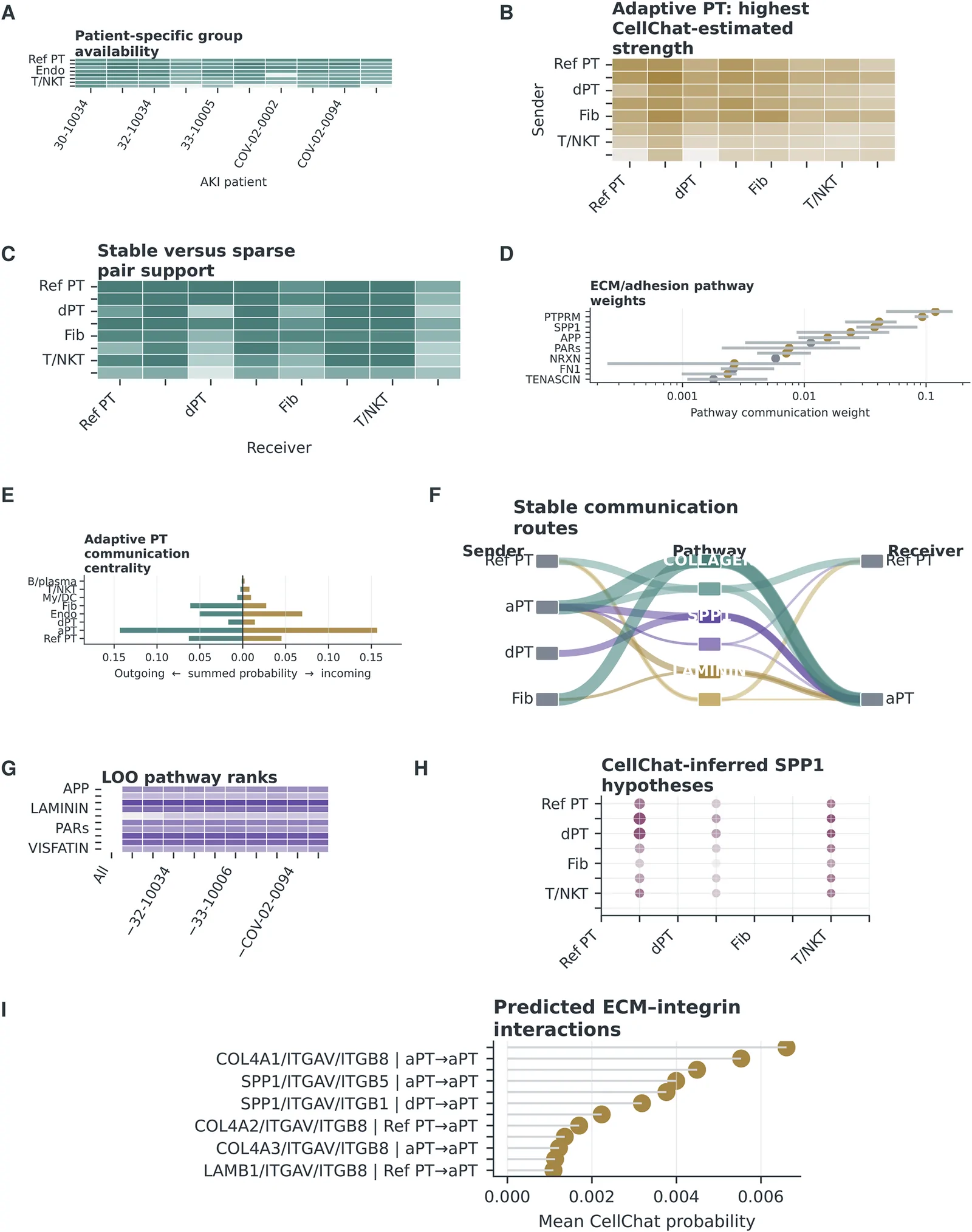
Patient-specific cell–cell communication identifies stable AKI tissue-context hypotheses. **(A)** Availability of communication groups in each AKI patient. **(B)** Median CellChat-estimated source–target strength. **(C)** Number of patients supporting each source–target pair. **(D)** Median pathway probabilities with patient-level intervals. **(E)** Incoming/outgoing centrality butterfly. **(F)** Stable source–target-pathway routes. **(G)** Leave-one-patient-out pathway-rank stability. **(H)** Recurrent SPP1 routes. **(I)** Extracellular-matrix-related ligand–receptor hypotheses. CellChat was run independently for each of the 10 AKI patients with CellChatDB.human, population-size adjustment, and a 20-cell minimum per group; consensus required inference in at least six patients.

### 3.8 Urinary single-cell analysis maps candidate accessibility and composition

Twenty-nine available urinary matrices passed through library-specific filtering, leaving 47,497 cells; starting barcode counts and retained-cell yields varied substantially across libraries (Figure 8A). Detected-gene counts, UMI abundance, and mitochondrial fractions also retained library-level dispersion after filtering, supporting the use of library-stratified rather than one global cutoff (Figure 8B). Reconstructed renal, immune, urogenital, and other proxies showed partial separation in the embedding (Figure 8C), whereas etiologies and individual versus pooled libraries occupied several overlapping regions (Figures 8D,E). The 27 analytic units were compositionally heterogeneous; primary summaries used 21 individual sample units from 13 patients, while six pools remained descriptive and were not treated as patients (Figure 8F). EPCAM/UMOD, PTPRC/CD3D, and KRT13/KRT5 patterns supported broad renal, immune, and urogenital proxy assignments, respectively, while fine identities remained unresolved (Figure 8G). Candidate reach differed depending on whether it was expressed per unit, renal-containing unit, all cells, or renal-proxy cells (Figure 8H). For ASPHD1, 59.3% of all units and 92.0% of renal-proxy units reached at least 1% detection; 1.91% of all cells and 5.14% of renal-proxy cells were positive, and 88.9% of positive cells mapped to the renal proxy. COL6A1-positive cells were 90.6% renal-derived, and COL7A1-positive cells were 74.9% renal-derived, whereas CMTM8 had only 52.4% renal and 33.1% urogenital contribution, and BRSK1 was predominantly immune-proxy (Figure 8I). COL6A1 and COL7A1 covered all three displayed etiologic strata more consistently; ASPHD1 covered two, and ENPP3 covered none at the same threshold (Figure 8J). Unit-level heatmaps confirmed non-uniform ASPHD1 and COL6A1 detection despite higher values in renal-proxy cells (Figure 8K). Reconstructed post-QC cell counts closely tracked published summaries (Spearman 0.9987; Pearson 0.9976), although P017.2 and P018.1 retained 950 and 340 additional cells, confirming close agreement for count recovery while leaving identities dependent on proxy markers (Figure 8L). The qualification plane placed ASPHD1 at moderate unit reach but high renal-source specificity, while COL6A1 combined higher reach with a similarly renal-biased source (Figure 8M).

**FIGURE 8:**
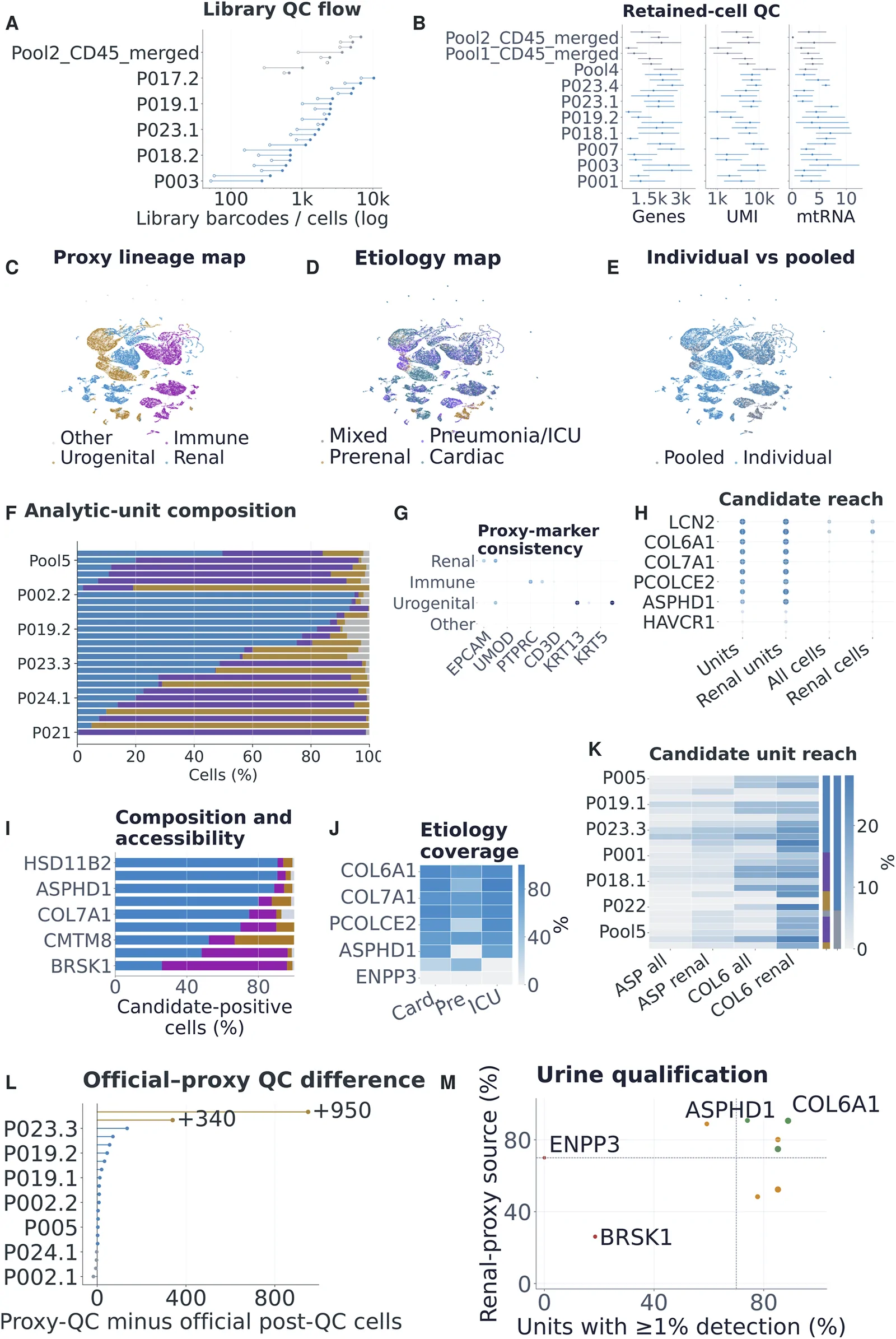
Urinary single-cell processing and candidate-accessibility audit. **(A)** Library-level quality-control flow. **(B)** Retained-cell quality distributions. **(C)** Reconstructed proxy-lineage embedding. **(D)** Etiology occupancy. **(E)** Individual-versus-pooled-unit occupancy. **(F)** Composition of the 27 analytic units, comprising 21 individual sample units from 13 patients and six pools; I denotes individual, and P denotes pool. **(G)** Proxy-marker consistency. **(H)** Candidate reach matrix. **(I)** Cellular source composition of candidate-positive cells. **(J)** Etiology coverage. **(K)** ASPHD1/COL6A1 detection heatmap across analytic units. **(L)** Official-summary-versus-proxy quality-control difference. **(M)** Urinary qualification plane. Cell identities were assigned using proxy markers; pooled units were summarized descriptively.

### 3.9 Tissue-state specificity and urinary accessibility were only partly aligned

Tissue-state specificity and urinary reach were only partly aligned: COL6A1 combined higher dPT specificity with broad urinary reach, whereas adaptive-associated ASPHD1 had moderate reach (Figure 9A). Ranks of the nine strict tissue candidates changed substantially in urine and were not correlated with tissue ranks (rho = −0.170, P = 0.663; Figure 9B). In the exploratory 13-patient analysis, the adaptive component correlated positively but inconclusively with the injury proxy (rho = 0.531, 95% CI -0.044 to 0.883, P = 0.062). The degenerative component correlated inversely (rho = −0.626, 95% CI -0.950 to −0.051, P = 0.022), and the degenerative-minus-adaptive contrast showed the strongest inverse association (rho = −0.725, 95% CI -0.947 to −0.223, P = 0.005; Figures 9C,D). Simultaneous adjustment for renal, immune, and urogenital proxy fractions preserved the inverse direction (partial rho = −0.774, 95% CI -0.986 to −0.079; Figure 9E), and leave-one-patient-out coefficients stayed negative from −0.818 to −0.650 (Figure 9F). ASPHD1, COL6A1, and contextual CMTM8 had distinct tissue, coverage, and renal-source profiles (Figure 9G), as did mature markers, including HAVCR1, LCN2, PODXL, HSD11B2, and GDF15 (Figure 9H).

**FIGURE 9:**
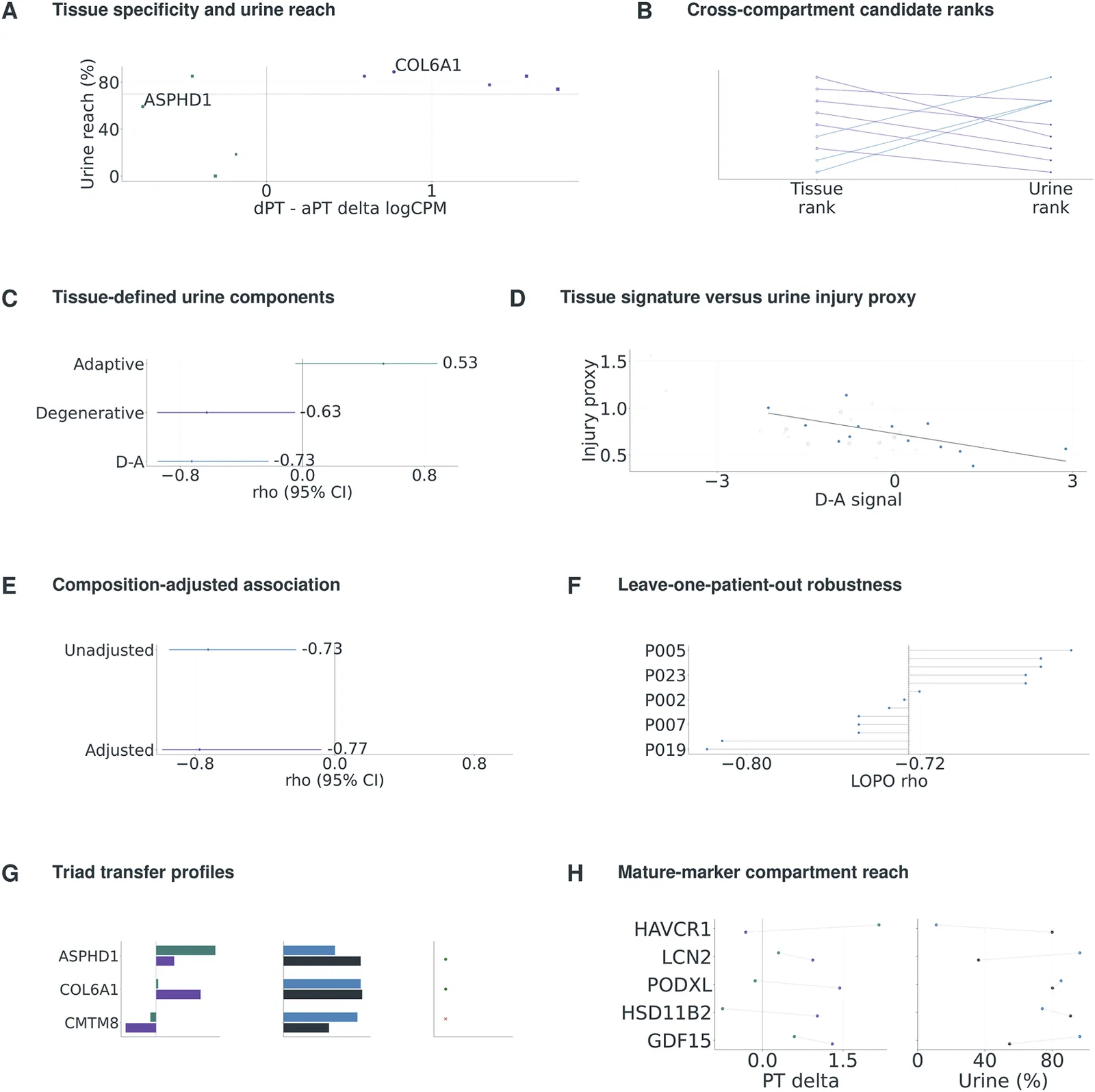
Tissue-state specificity and urinary accessibility are only partly aligned. **(A)** Tissue-state effect versus urinary reach; circles indicate strict tissue replication, squares indicate non-strict context, and point size denotes renal-proxy share. **(B)** Tissue and urine candidate-rank flow for the nine strict candidates (Spearman rho = −0.170, P = 0.663). **(C)** Tissue-defined urinary components versus the injury proxy in 13 patients; points are patient aggregates, and intervals are from 5,000 patient bootstrap resamples. **(D)** Patient-level degenerative-minus-adaptive signal versus injury proxy (rho = −0.725, P = 0.005). **(E)** Association before and after simultaneous adjustment for renal, immune, and urogenital proxy fractions. **(F)** Leave-one-patient-out coefficients (n = 12 each; range −0.818 to −0.650). **(G)** ASPHD1/COL6A1/CMTM8 transfer profiles; CMTM8 is a context gene, not a strict dual-tissue replicate. **(H)** Established-marker transfer profiles. Tissue and urine cohorts are unpaired; patients are the primary biological units.

### 3.10 Longitudinal qualification resolved transplant trajectories and candidate trade-offs

Serial urinary ASPHD1 varied bidirectionally across six patients rather than following a common monotonic post-AKI pattern (Figure 10A). First-to-last adaptive, degenerative, and degenerative-minus-adaptive changes were also bidirectional; for example, P23 had an adaptive change of +2.48 and a contrast change of −3.09, whereas P17 had values of −1.76 and +1.64 (Figure 10B). No adjudicated recovery endpoint was available, so these urine trajectories were descriptive. In 37 transplant recipients with all four biopsy time-points, ASPHD1 changed neither acutely (POST-PRE delta 0.0278, FDR 0.478) nor persistently (12-month-PRE delta −0.0113, FDR 0.841; Figure 10C). GDF15 served as a responsive benchmark: it increased acutely by 2.381 (FDR 3.99 x 10^−10^) and fell below baseline at 12 months by −0.680 (FDR 4.38 x 10^−5^; Figure 10D). Among the six displayed genes, CMTM8, HSD11B2, and PODXL decreased acutely, with FDR values of 0.0213, 0.00610, and 0.000756, respectively; COL6A1, PODXL, and GDF15 decreased at 12 months, with FDR values of 0.0250, 0.0110, and 4.38 x 10^−5^ (Figure 10E). Acute and persistent effects did not align on a single diagonal, showing that early direction did not predict 12-month direction (Figure 10F). In the non-weighted qualification plane, ASPHD1 had adaptive-PT direction consistency in 100% of patients but 59.3% urine-unit coverage; COL6A1 had 88.9% coverage and 90.6% renal source but only 20% adaptive-direction consistency; COL7A1 had the largest tissue-effect floor, 85.2% coverage, 74.9% renal source, and 60% adaptive-direction consistency (Figure 10G). Two thousand bootstrap iterations preserved these tradeoffs (Figure 10H): ASPHD1 had a 99% rank-1 frequency for PT direction, COL6A1 ranked first for urine coverage and renal source in 63% and 81% of iterations, and COL7A1 ranked first for tissue-effect floor in 97% (Figure 10I). Pareto inclusion was 100%, 100%, and 99.8%, respectively. ASPHD1 advanced to functional testing because its dual-tissue replication, reproducible adaptive localization, urinary detectability, and intracellular assayability best matched the experimental question across these non-weighted objectives.

**FIGURE 10:**
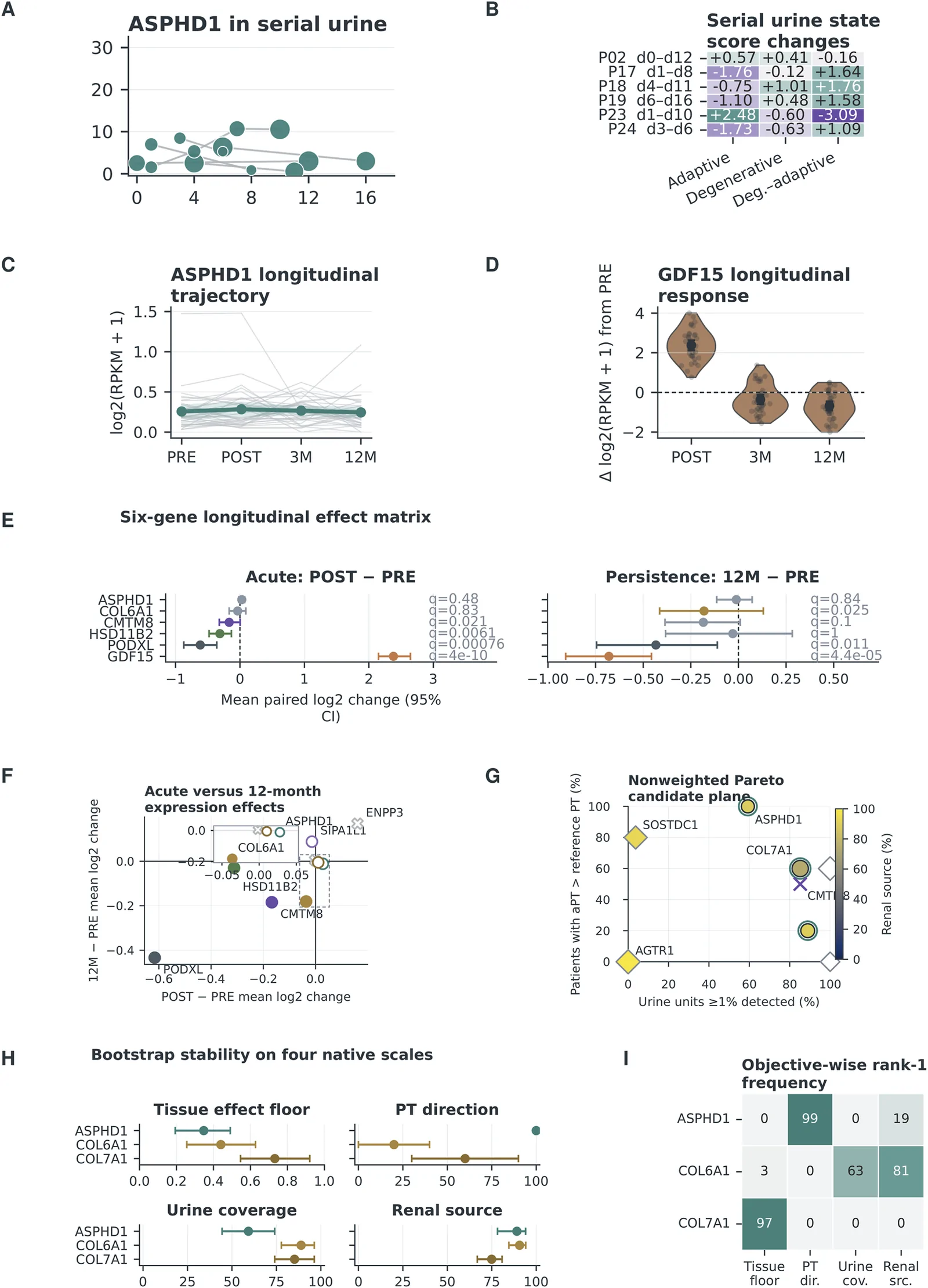
Longitudinal qualification distinguishes transplant trajectories and candidate trade-offs. **(A)** Serial urinary ASPHD1 in six patients; no adjudicated recovery endpoint was available. **(B)** First-to-last adaptive, degenerative, and degenerative-minus-adaptive urinary-score changes. **(C)** ASPHD1 trajectory among the 37 GSE126805 recipients with all four transplant-biopsy time-points. **(D)** GDF15 longitudinal response. **(E)** Six-gene acute and persistent paired-effect intervals. **(F)** Acute-versus-12-month effect plane. **(G)** Non-weighted Pareto qualification; ASPHD1, COL6A1, and COL7A1 are eligible, while CMTM8 remains a context gene. **(H)** Confidence intervals from 2,000 patient bootstrap resamples for the native-scale qualification objectives. **(I)** Bootstrap rank frequencies. GSE126805 paired contrasts used two-sided Wilcoxon tests with Benjamini–Hochberg correction over each displayed gene family. Serial urine panels are descriptive; transplant analyses concern ischemia–reperfusion trajectories.

### 3.11 Cisplatin induces ASPHD1 and exposes a viability phenotype after silencing

After 24 h, CCK-8 signal declined progressively from 5 to 40 μM cisplatin; 10 μM retained approximately three-quarters of the displayed vehicle value, whereas 20 and 40 μM produced more severe loss (Figure 11A). HAVCR1 was unchanged at 5 μM but increased at 10, 20, and 40 μM (Figure 11B). On that basis, 10 μM for 24 h was selected to induce injury while preserving a recovery window. ASPHD1 mRNA was higher at the exposure endpoint and after 24 and 72 h of recovery than in the matched vehicle groups, with the largest displayed increase at R24 (Figure 11C). Representative R24 microscopy showed stronger ASPHD1 fluorescence after cisplatin (Figure 11D), and quantified intensity increased across the three independent experiments (Figure 11E). CCK-8 signal increased during recovery in both the vehicle and cisplatin groups and remained lower after cisplatin at the matched time-points (Figure 11F). Both siASPHD1-1 and siASPHD1-2 reduced ASPHD1 transcript abundance, with the deeper reduction produced by siASPHD1-2 (Figure 11G). Neither siRNA materially changed the displayed vehicle CCK-8 values, but both further reduced the signal after cisplatin, indicating an injury-context-dependent vulnerability (Figure 11H). Under vehicle, HAVCR1, COL6A1, and CMTM8 showed little siRNA effect. After cisplatin, both siRNAs increased HAVCR1 and COL6A1 and further reduced CMTM8 relative to siNC (Figures 11I–K). Both siRNAs reproduced the cisplatin-associated viability and injury-marker phenotype.

**FIGURE 11:**
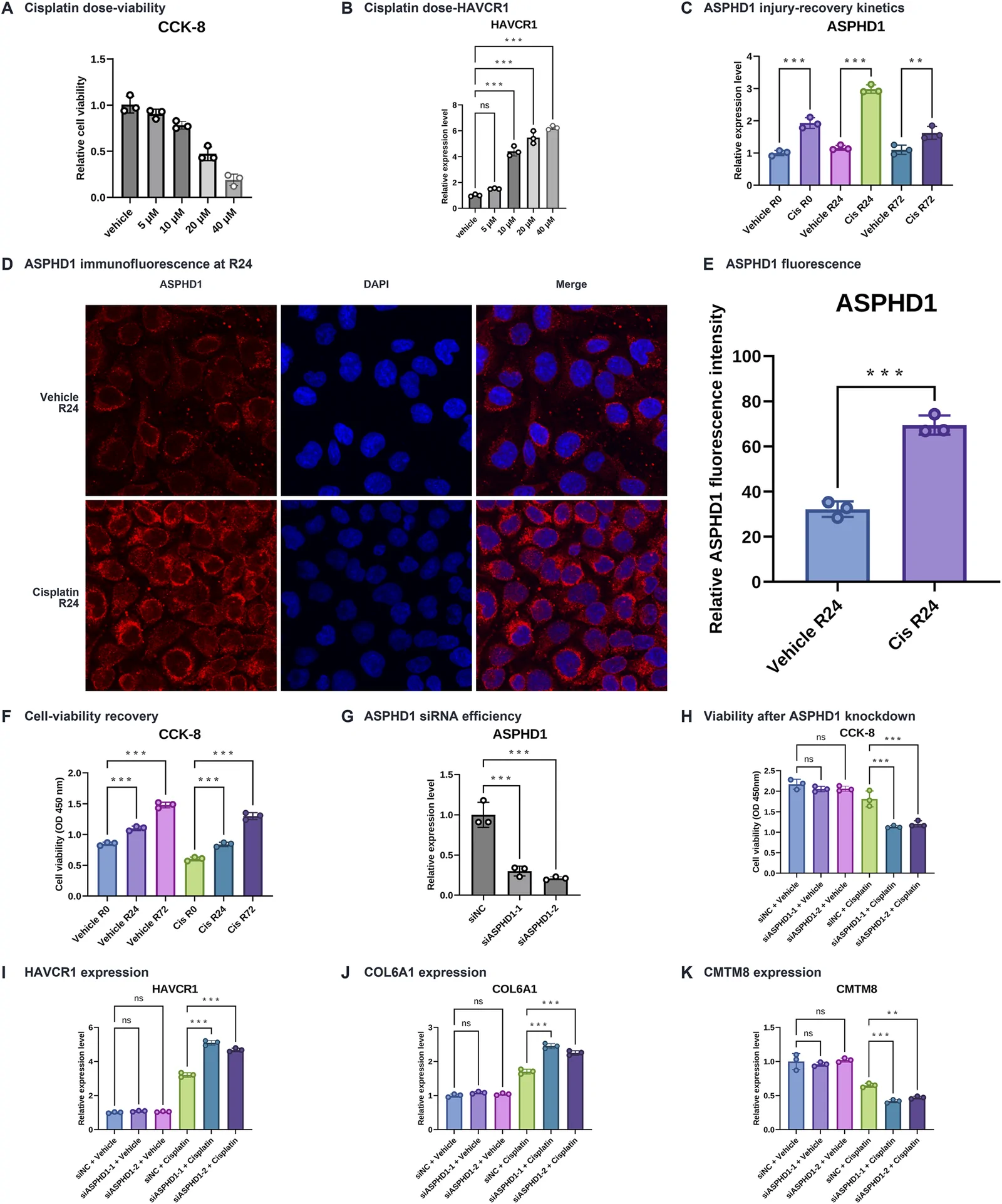
Cisplatin induces ASPHD1, and ASPHD1 silencing compromises post-injury recovery. **(A)** RPTEC/TERT1 viability after 24-h cisplatin exposure. **(B)** HAVCR1 induction across cisplatin doses. **(C)** ASPHD1 mRNA during injury and recovery. **(D)** Representative ASPHD1 immunofluorescence at R24. **(E)** ASPHD1 fluorescence quantification. **(F)** Viability during recovery after cisplatin washout. **(G)** ASPHD1 mRNA after transfection with siNC, siASPHD1-1, or siASPHD1-2. **(H)** Viability after ASPHD1 silencing with vehicle or cisplatin. **(I–K)** HAVCR1, COL6A1, and CMTM8 expression under the same conditions. Points represent three independent biological experiments; bars show the mean ± SD. Statistical tests and multiple-comparison procedures are described in Methods, and significance symbols are defined in the panels. Knockdown was verified at the mRNA level.

### 3.12 ASPHD1-silencing increases caspase-associated apoptosis

Representative annexin V-APC/PI plots showed very low apoptotic populations under the vehicle in the siNC, siASPHD1-1, and siASPHD1-2 groups. Cisplatin expanded the apoptotic quadrants in siNC, and both ASPHD1 siRNAs produced a further expansion (Figure 12A). Across three independent experiments, the total apoptotic fraction remained near baseline and was not different among vehicle groups, whereas cisplatin increased it to approximately 10% with siNC and approximately 40% with either siASPHD1 sequence (Figure 12B). The deeper siASPHD1-2 experiment showed no comparable transcript shift under vehicle; after cisplatin, BAX and BBC3 increased further and BCL2 decreased relative to siNC (Figure 12C). z-VAD-FMK had little effect on the displayed siNC/cisplatin viability but partially restored viability in the siASPHD1-2/cisplatin group (Figure 12D). Intracellular flow cytometry provided an independent caspase readout: representative cleaved-caspase-3 positivity was approximately 1.6%–2.2% under vehicle, 23% after siNC/cisplatin, and 55% after siASPHD1-2/cisplatin (Figure 12E), and replicate-level quantification confirmed the increase from roughly 20%–50% after silencing (Figure 12F). Together, the two-siRNA annexin phenotype, apoptosis-related transcript changes, cleaved-caspase-3 increase, and partial z-VAD-FMK rescue indicate a caspase-associated vulnerability after ASPHD1-silencing.

**FIGURE 12:**
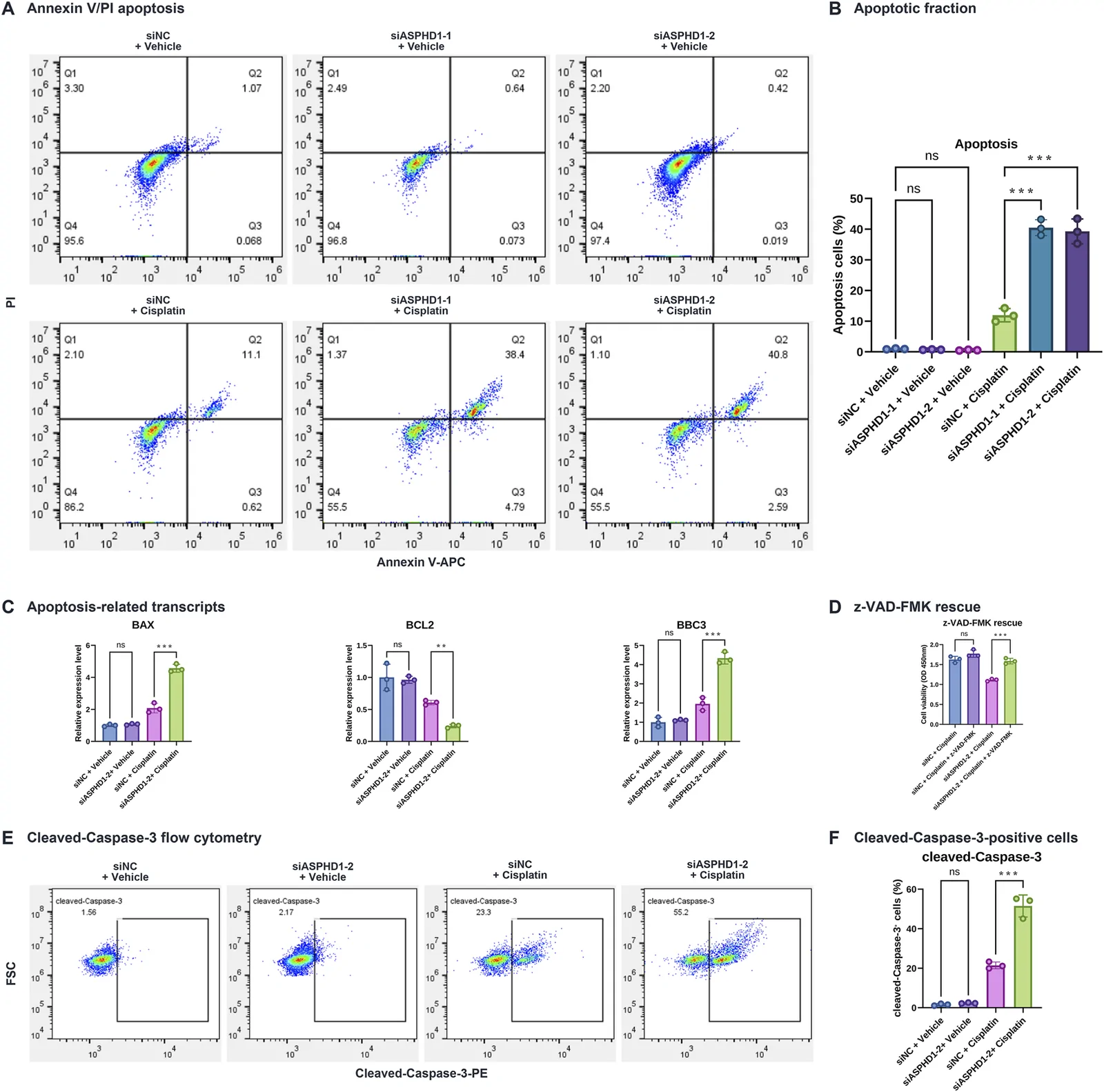
ASPHD1-silencing increases caspase-associated apoptosis after cisplatin injury. **(A)** Representative annexin V-APC/propidium iodide flow-cytometry plots. **(B)** Total apoptotic fraction for siNC, siASPHD1-1, and siASPHD1-2 under vehicle or cisplatin. **(C)** BAX, BCL2, and BBC3 transcript abundance in the siNC/siASPHD1-2 experiment. **(D)** Cell viability after z-VAD-FMK rescue. **(E)** Representative cleaved-caspase-3 flow-cytometry plots. **(F)** Cleaved-caspase-3-positive fraction. Data are the mean ± SD from three independent biological experiments. Statistical tests are described in Methods. Both independent siRNAs were used for the initial apoptotic phenotype; siASPHD1-2 was used for the deeper expression, caspase-3, and rescue assays.

### 3.13 ASPHD1-silencing increases lipid peroxidation and ferroptosis-associated vulnerability

siASPHD1-2 did not materially alter SLC7A11, ACSL4, or PTGS2 under the vehicle; after cisplatin, however, SLC7A11 decreased further, while ACSL4 and PTGS2 increased relative to siNC (Figure 13A). Representative C11-BODIPY plots showed little vehicle difference but a larger oxidized population after siASPHD1-2/cisplatin (Figure 13B). Quantification across three experiments placed the oxidation-positive fraction at approximately 20%–25% with siNC/cisplatin and 45%–50% after ASPHD1-silencing (Figure 13C). GPX4 immunofluorescence was similar between the vehicle groups, lower after cisplatin, and lower still in the siASPHD1-2/cisplatin condition (Figure 13D); the mean fluorescence intensity confirmed the additional decrease after silencing (Figure 13E). Ferrostatin-1 did not materially alter the displayed viability of siNC/cisplatin cells but partially restored viability in the siASPHD1-2/cisplatin group (Figure 13F). The same context dependence was evident for lipid peroxidation: representative plots shifted toward fewer oxidized cells after ferrostatin-1 (Figure 13G), and quantification showed no significant change in siNC/cisplatin but a reduction from approximately 50%–30% in the siASPHD1-2/cisplatin group (Figure 13H). Convergent transcript, C11-BODIPY, GPX4-fluorescence, and selective rescue findings support increased ferroptosis-associated vulnerability in this model.

**FIGURE 13:**
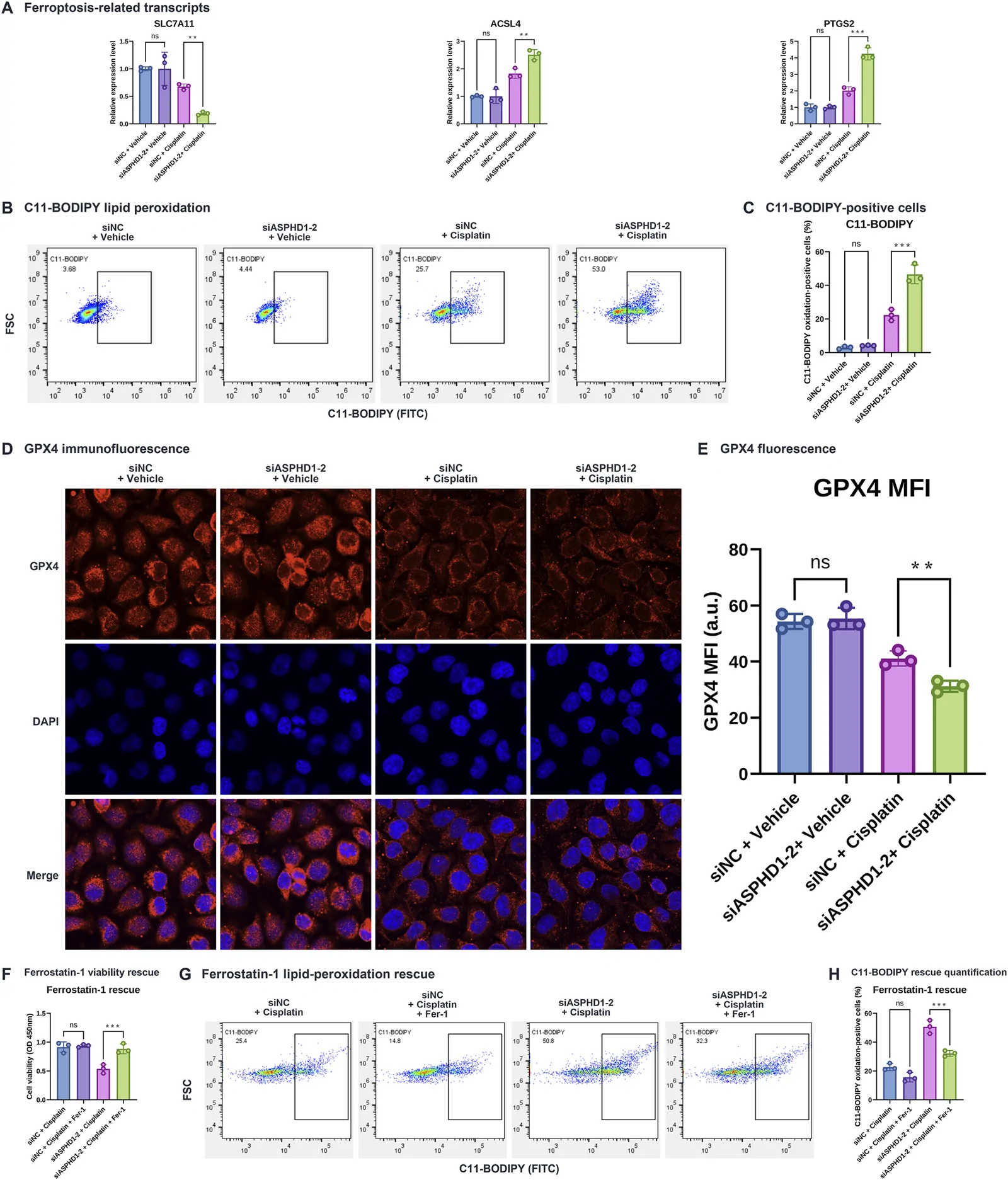
ASPHD1-silencing increases lipid peroxidation and ferroptosis-associated vulnerability. **(A)** SLC7A11, ACSL4, and PTGS2 transcript abundance after siNC or siASPHD1-2 with vehicle or cisplatin. **(B)** Representative C11-BODIPY flow-cytometry plots. **(C)** C11-BODIPY oxidation-positive fraction. **(D)** Representative GPX4 immunofluorescence. **(E)** GPX4 fluorescence quantification. **(F)** Viability after Fer-1 rescue. **(G)** Representative C11-BODIPY plots after Fer-1. **(H)** Lipid-peroxidation quantification after Fer-1. Points represent three independent biological experiments; bars show the mean ± SD. Statistical tests are described in Methods.

## 4 Discussion

Independent tissue replication placed ASPHD1 within a reproducible AKI-associated set, patient-resolved pseudobulk localized it preferentially to adaptive PT, and the four death-program scores separated across the reference–aPT–dPT ordering: apoptosis-associated enrichment extended across both injured states, whereas ferroptosis-associated enrichment was strongest in dPT. Injured PT cells occupy heterogeneous adaptive, inflammatory, cycling, and failed-repair configurations rather than a single terminal identity, and experimental and human multi-omic studies have identified structured failed-repair programs with distinct candidate drivers (Kirita et al., 2020; Ledr et al., 2024). The adaptive localization of ASPHD1 is, therefore, biologically informative, although the cross-sectional design cannot resolve whether aPT precedes dPT or whether ASPHD1 influences conversion between them. Regulated death pathways share upstream stress signals and protective systems, and transcriptional enrichment differs from direct evidence of membrane rupture, protease activation, or death execution (Sanz et al., 2023; Maremonti et al., 2022). Together, the human data describe a state-organized transcriptional landscape and nominate ASPHD1 as an adaptive-state stress candidate.

The cell experiments addressed whether reducing ASPHD1 changes tolerance to cisplatin injury. Two independent siRNAs reproduced the initial loss of viability and increase in annexin V positivity, while the deeper siASPHD1-2 series showed higher BAX and BBC3, lower BCL2, more cleaved caspase-3, and partial rescue by z-VAD-FMK. A separate set of readouts—greater C11-BODIPY oxidation, reduced GPX4 fluorescence, lower SLC7A11, higher ACSL4 and PTGS2, and selective partial rescue by ferrostatin-1—supported increased lipid-peroxidation vulnerability. Ferroptosis is defined by iron-dependent phospholipid peroxidation and the failure of antioxidant defenses, with GPX4 serving as a central suppressive system; kidney tubular susceptibility increases when ferroptosis-surveillance mechanisms are impaired (Stockwell et al., 2017; Tonnus et al., 2021). Apoptotic and regulated-necrotic pathways can intersect through shared redox stress, mitochondrial injury, and inflammatory amplification, so partial rescue by a pathway inhibitor does not prove one exclusive death mechanism (Sanz et al., 2023; von Massenhausen et al., 2018). GPX4 biology is consistent with the fluorescence result but does not demonstrate a direct ASPHD1–GPX4 interaction (Yang et al., 2014). Cisplatin nephrotoxicity also involves tubular, inflammatory, vascular, and metabolic processes (Pabla and Dong, 2008; Ozkok and Edelstein, 2014). Thus, ASPHD1-silencing lowers cellular tolerance and reveals caspase-associated and ferroptosis-associated components in this model.

Urinary accessibility varied with cellular composition. Urine contains variable mixtures of renal, immune, and urogenital cells, and single-cell studies show that the recovered composition determines which kidney signals can be observed in a specimen (Abedini et al., 2021). ASPHD1-positive cells were predominantly renal-proxy, but coverage varied across units and etiologic strata; tissue rank did not predict urine rank, and the strongest urinary state-score association came from unpaired cohorts with reconstructed proxy identities. Biomarker development requires a defined clinical task, such as risk assessment, diagnosis, etiologic classification, persistence, or recovery, because evidence for one use cannot be transferred automatically to another (Schrezenmeier et al., 2017; Ostermann et al., 2020; Murray et al., 2014). Prospective multicenter studies relating predefined urinary measurements to adjudicated outcomes illustrate the evidentiary distance between transcript nomination and diagnostic or prognostic validation (Parikh et al., 2011). Here, the cellular-source and composition audit informs assay design, but the absence of healthy urinary controls, matched tissue–urine pairs, an analytical threshold, and an outcome endpoint precludes claims of transport, discrimination, prognosis, or recovery. An independent cohort should measure ASPHD1 RNA and, if a validated assay becomes available, protein in phenotyped urine samples with predefined endpoints and comparison against established injury and persistence markers.

The main limitations are the different disease and reference contexts of the tissue cohorts, the 10-patient PT-state analysis, reconstructed urinary identities, descriptive pooled urine samples, and use of a transplant series that cannot represent general-AKI recovery. Molecularly distinct AKI sub-phenotypes with different outcomes and treatment responses have been reported, reinforcing the need to connect a state marker to a defined population and clinical decision (Bhatraju et al., 2019; Rodrigues and Endre, 2023). RPTEC/TERT1 provides a reproducible proximal-tubule model and has precedent in cisplatin multi-omic toxicology, but an immortalized monolayer incompletely represents *in vivo* kidney injury (Wilmes et al., 2015). The experiments comprised three independent biological replicates, ASPHD1 depletion was confirmed at mRNA rather than protein level, and the deeper assays used one siRNA. Protein-level knockdown verification, sequence-independent rescue by ASPHD1 re-expression, direct target or interactome assays, and validation in patient-derived or more physiologically structured PT systems are, therefore, priorities before clinical biomarker testing.

## 5 Conclusion

Patient-resolved human data indicate that regulated cell-death-associated transcriptional programs are organized across AKI PT states rather than uniformly activated: apoptosis-associated enrichment spans adaptive and degenerative states, whereas ferroptosis-associated enrichment is the strongest in the degenerative state. ASPHD1 is a reproducible adaptive-state candidate whose silencing increases cisplatin-associated apoptotic and ferroptosis-related vulnerability in RPTEC/TERT1 cells.

## Data Availability

The original contributions presented in the study are included in the article/supplementary material; further inquiries can be directed to the corresponding authors.
